# The cognitive nuances of surprising events: exposure to unexpected stimuli elicits firing variations in neurons of the dorsal CA1 hippocampus

**DOI:** 10.1007/s00429-018-1681-6

**Published:** 2018-05-22

**Authors:** Ornella Valenti, Nace Mikus, Thomas Klausberger

**Affiliations:** 1 Division of Cognitive Neurobiology, Centre for Brain Research, Medical University Vienna, Spitalgasse 4, 1090 Vienna, Austria; 2 Division of Neurophysiology and Neuropharmacology, Centre for Physiology and Pharmacology, Medical University Vienna, Schwarzspanierstrasse 17, 1090 Vienna, Austria; 3 Department of Basic Psychological Research and Research Methods, University of Vienna, Vienna, Austria

**Keywords:** Match-mismatch learning, Hippocampal neurons, Virtual reality, Novelty detection, Reward valence, Mice

## Abstract

The ability to recognize novel situations is among the most fascinating and vital of the brain functions. A hypothesis posits that encoding of novelty is prompted by failures in expectancy, according to computation matching incoming information with stored events. Thus, unexpected changes in context are detected within the hippocampus and transferred to downstream structures, eliciting the arousal of the dopamine system. Nevertheless, the precise locus of detection is a matter of debate. The dorsal CA1 hippocampus (dCA1) appears as an ideal candidate for operating a mismatch computation and discriminating the occurrence of diverse stimuli within the same environment. In this study, we sought to determine dCA1 neuronal firing during the experience of novel stimuli embedded in familiar contexts. We performed population recordings while head-fixed mice navigated virtual environments. Three stimuli were employed, namely a novel pattern of visual cues, an odor, and a reward with enhanced valence. The encounter of unexpected events elicited profound variations in dCA1 that were assessed both as opposite rate directions and altered network connectivity. When experienced in sequence, novel stimuli elicited specific responses that often exhibited cross-sensitization. Short-latency, event-triggered responses were in accordance with the detection of novelty being computed within dCA1. We postulate that firing variations trigger neuronal disinhibition, and constitute a fundamental mechanism in the processing of unexpected events and in learning. Elucidating the mechanisms underlying detection and computation of novelty might help in understanding hippocampal-dependent cognitive dysfunctions associated with neuropathologies and psychiatric conditions.

**Electronic supplementary material:**

The online version of this article (10.1007/s00429-018-1681-6) contains supplementary material, which is available to authorized users.

## Introduction

Detection of novelty is essential for survival: The experience of novel events triggers a cascade of brain alterations that leads to enhanced attention, learning, and memory. Increasing evidence indicates that not novelty per se, but rather the ability to match expected events to encountered stimuli is fundamental in this function (Jenkins et al. 2004; Lisman and Grace 2005; Kumaran and Maguire 2007a; Poppenk et al. 2010). The dopamine system and the ventral tegmental area (VTA) are essential during the computation of novel stimuli (Ljungberg et al. 1992; Bunzeck and Düzel 2006). Nevertheless, detection of novelty might not be operated by the VTA. Indeed, the arousal of the dopamine system might represent a secondary effect, driven by detection of novelty in upstream structures (Legault and Wise 2001; Lisman and Grace 2005; Kumaran and Maguire 2009). Hippocampus is engaged in the encoding and retrieval of experiences and might operate a comparison between the consistency of expected and perceived events (Vinogradova 1984; Hasselmo and Wyble 1997). In this respect, if an event is aligned with a subject expectation, changes in hippocampal activity induce memory retrieval or pattern completion. Conversely, a mismatch between encountered stimuli and stored episodes might favor pattern separation and learning (Hasselmo and Schnell 1994; Davachi 2006; Eichenbaum et al. 2007; Squire et al. 2007). Among all subfields, the dorsal CA1 hippocampus (dCA1) appears to host properties consistent with a comparator function (Vinogradova 1984; Hasselmo et al. 2002; Kumaran and Maguire 2006a). Evidence from rodents provides support to the pivotal role of the dCA1 in match/mismatch learning (Vinogradova 2001; Fyhn et al. 2002; Jenkins et al. 2004). When examined in models based on expected vs. unexpected conditioned stimuli, novelty-evoked dCA1 responses were observed within 100-msec (Ruusuvirta et al. 1995; Brankačk et al. 1996; Grunwald et al. 1998); such a brief interval would indeed reflect detection of novelty within dCA1. Likewise, imaging studies reported a maximal level of responses when the employed stimuli were not entirely novel, but retained elements of previous experiences (Köhler et al. 2005a; Kumaran and Maguire 2006b, 2009; Duncan et al. 2012a).

Pyramidal cells of the dCA1 are long projecting neurons and, through the subiculum, carry contextual information to downstream structures, including the VTA (Floresco et al. 2001, 2003; Grace et al. 2007). Within this group, place cells exhibit discriminatory firing in association to specific places of the environment (O’Keefe and Dostrovsky 1971), foster the inner representation of space (Dragoi and Tonegawa 2013), and change their activity during the experience of novelty (Wilson and McNaughton 1993; Fyhn et al. 2002; Anderson and Jeffery 2003; Frank et al. 2004; Leutgeb et al. 2004; Jackson and Redish 2007; Kelemen and Fenton 2010; Jezek et al. 2011). GABAergic interneurons innervate principal cells at specific sub-cellular domains and equip their targets with the tuned release of GABA (Klausberger and Somogyi 2008). Therefore, interneurons orchestrate principal cell firing and set the stage for synchronized activity during network oscillations. Previous reports indicate that interneurons also alter their activity in face of novel events (Wilson and McNaughton 1993; Frank et al. 2004; Nitz and McNaughton 2004; Assisi et al. 2011; Dupret et al. 2013).

In this study, we aimed at demonstrating dCA1 neuronal firing during sequential exposure to novel stimuli delivered in otherwise familiar contexts. To this end, we recorded dCA1 population activity while mice navigated virtual mazes. Three stimuli were employed, namely novel visual cues, odor, and unexpected rewards, which were typically presented within one specific area of the virtual corridor. This design allowed elucidating: (1) the means by which a given dCA1 neuron signals the occurrence of stimuli holding different properties; (2) whether a pattern emerges among all the evoked responses; (3) whether novel stimuli, experienced subsequentially within the same context and in restricted time windows, induce response cross-sensitization. Moreover, the analysis of the event-triggered responses aided in demonstrating the locus of novelty detection within hippocampus.

## Experimental procedures

### Subjects and head-plate implantation surgeries

All procedures, handling, and experiments of animals were conducted in accordance with international regulations and ethical standards as well as with the regulations of the Medical University of Vienna and were performed under the licenses approved by the Austrian Ministry of Science.

Data from 11 C57BL/6 adult male mice (28–36 g) were included in this study. Subjects were grown from an in-house colony and housed accordingly to international/well-established standards. Implantation surgeries were conducted as previously described (Lasztoczi and Klausberger 2016). Mice were anesthetized with isoflurane (Forane, AbbVie; induction) and mounted on a stereotaxic frame (Kopf instrument). Two screws were implanted above the cerebellum, serving as ground and reference, respectively. Two additional screws were driven above the prefrontal cortex. Supported by the four screws, a custom-made plastic head plate (Mr. Asenov; IST, Vienna) was implanted. Coordinates aiming at the right dorsal hippocampus (lateral, 1.3 mm; posterior, 1.9 mm; Franklin and Paxinos 2008) were calculated, and the skull above was marked for future reference. Mice were injected with Metamizole (15 µl Novalgin, Sanofi; 25 mg /100 g body weight) and singly housed.

### Training of head-fixed mice during navigation in a virtual environment

**Fig. 1:**
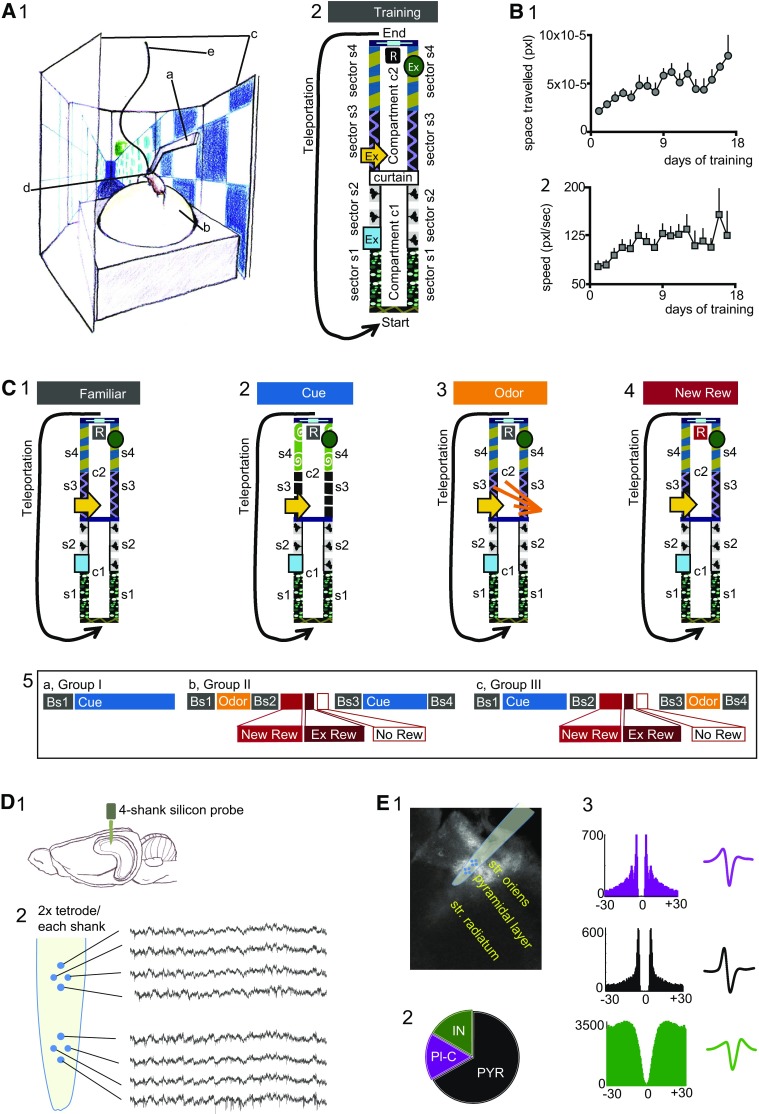
Behavioral set-up and dCA1 population recordings. **A** 1 Training and/or recording configuration (PhenoSys JetBall): a mice were head-fixed to a metallic bar, in turns connected to a stereotaxic frame, b air-suspended/foaming sphere, c six-screen system surrounding the subjects, d needle providing the rewards, e cable of the silicon probe. 2 Drawing of the training corridor; view from the top. *Ex* extra-corridor cues, *R* reward location. Note the different patterns in wall cues among the four sectors, s1–s4, and the curtain separating the corridor into two compartments. **B** Training curves 1 average space traveled during navigation of the corridor; 2 mice velocity. **C** 1 The corridor employed during recordings of baseline trials (familiar) was identical to the training corridor, 2 Novel cue corridor; note the cue-replacements on the walls of sectors s3 and s4. 3 Odor corridor, odor delivery in s3 is indicated by yellow lines. 4 Delivery of the unexpected rewards (red box) during reward trials occurred at the same location as the familiar reward. 5 Top- schematic representation of the experimental protocols; note the alternated sequence of baselines and novelty trials and the inverted order of stimulus-presentation between group II and III. Baseline (Bs) = grey color; novel cues = blue; odor = yellow; novel reward = red. Bottom- Sequence of the three reward protocols; NewRew = novel reward, ExRew = extra reward, NoRew = no reward trials. **D** 1 Silicon probe placement in respect to the mouse brain during tetrode recordings. 2 Left- drawing of one shank of the silicon probe; note the contacts in tetrode configuration. Right-field potentials and embedded units recorded for one experiment. **E** 1 Image of post hoc histological analysis for one experiment illustrates the location of one shank in respect to the different layers of the dCA1. 2 Distribution of the different neuronal classes recorded; 3 examples of autocorrelograms and spike waveforms for: place cells (Pl-C, purple; *n* = 32 cells), pyramidal neurons (PYR, black; *n* = 128 cells), and interneurons (IN, green; *n* = 32 cells)

Subjects were randomly assigned to one experimental protocol; at reaching of the pre-surgical weight, they were handled daily and water-restricted (1-ml water per day). After 3–5 days, mice were fixed to a metallic support through the 4-screw-anchored plate (Fig. 1A1-a), which was in turns connected to a stereotaxic apparatus (Kopf Instruments). Although head-fixed, subject movements were fully allowed on an *X*–*Y* plane through an air-suspended Styrofoam ball (Fig. 1A1-b) positioned inside a 6-screen-TFT monitor-system (Fig. 1A1-c; PhenoSys JetBall). The movements of the ball were captured by an infrared sensor and converted into movements of the images projected onto the screens, (Fig. 1A1-b, c) to ultimately reproduce navigation in virtual environments (VR). All experimental protocols were designed and controlled by the PhenoSoft Control software. Mice were trained to run progressively longer corridors, the dimension of the final corridor being of 2400 pixels (pxl). The virtual corridors presented four distinct cue patterns on the internal walls, which differed along the longitudinal line but were identical between the right and left walls (Fig. 1A2). This configuration aided at visually dividing the corridor into four sectors (600pxl each), termed sectors s1 to s4 (Fig. 1A2). All visual cues were chosen in different shapes, luminosity, and also colors but within the range of wavelengths perceived by the mouse visual system (Bridges 1959; Jacobs et al. 2004). The reward area and the starting position were also identified by distinct cues (Fig. 1A2). Additional 3-D images were inserted on top of the corridor walls (extra-corridor cues, Ex) and served to facilitate mice navigation within the maze (Fig. 1A2). A virtual square image (termed curtain) was inserted in the middle of the maze to further separate the corridor into two compartments, i.e. compartment c1 (containing sectors s1 and s2) and c2 (s3 and s4; Fig. 1A2). At the end of the corridor (Fig. 1A2), a reward, consisting of 1% sucrose solution, was provided (Fig. 1A1-d). Following reward consumption, mice spent additional 3-sec with screens off, a period that helped in discriminating the end of one lap and the beginning of the next. Mice were then teleported to the starting position (Fig. 1A2) to begin a new trial; trials were repeated in loops. Noticeably, teleportation allowed resembling a procedure typically operated in freely-moving studies, in which rodents are physically relocated to the beginning of the maze. Throughout the training, the amount of reward was decreased to 3 µl/trial, and this same amount was used during electrophysiology recordings. Mice became progressively acquainted also with the activation of a 2-way solenoid valve, which, during odor experiments, provided an odor essence. The confidence of operating the navigation system, maze exploration and mice velocity were also monitored (Fig. 1B1, 2). As mice reliably reached the ability to perform the number of trials for the assigned protocol, they were prepared for craniotomy (Forro et al. 2013). Briefly, a restricted window above dCA1 was drilled, the dura was gently removed and the surgical area was protected with silicon (Kwik-Cast, World Precision Instruments). Following ~ 12-h, electrophysiology recordings of dCA1 neuron activity were performed coincidentally to the execution of the assigned protocol (Fig. 1C).

### A novel hippocampal-based match/ mismatch tasks for the detection of novelty

The impact of unexpected events on dCA1 neuronal firing was assessed by employing a hippocampus-dependent match/mismatch task. The use of virtual mazes allowed deliveries of the novel stimuli in discrete locations and time intervals. Three novel stimuli were selected, and consisted of: (1) a novel pattern of visual cues, (2) an odor, and (3) a reward with enhanced valence. All stimuli were chosen on the basis of shared proprieties/mechanisms (i.e. sensory stimuli *vs*. incentive salient stimuli) and in accordance with previous reports suggesting their role in different types of learning (i.e. observation-driven *vs*. association). Novel stimuli were presented one per time in properly designed corridors (Fig. 1C1–4). With respect to the corridor employed during training (i.e. familiar corridor), novel corridors maintained the geometry and retained all extra-maze cues as well as the cue-patterns at the starting and the reward area. Compartment c1 of the novel corridor was also kept identical with respect to the familiar corridor; in contrast, compartment c2 was properly modified to allow the investigation of novel stimuli (Fig. 1C1–4). Thus, in novel corridors, unexpected events resulted embedded in environments preserving a familiar context. Although did not hamper mouse navigation, the curtain, located in the middle of the corridor, provided an optical impediment and hid the compartment c2 from the starting position, allowing the experience of novel stimuli only after crossing into the novel compartment (Fig. 1C1–4). Novelty in visual stimuli was achieved by replacing the cue-patterns on sector s3 and s4 walls (Fig. 1C2). For odor experiments, an odor was released through a MATLAB-controlled valve (Bio-Chem Fluidics) in s3 at mice crossing of the curtain (Fig. 1C3). Vanilla (Schlaraffen GmbH) was selected based on preliminary behavioral observations, as this essence induced approach/neutral behavior in contrast, for example, to peppermint. To elucidate whether dCA1 neurons might also signal the experience of unexpected rewards, we employed a potentially more likable and novel reward. The novel reward consisted of an apricot and peach syrup (Yo Austria) diluted to 10% sucrose concentration (i.e.10× concentration of the familiar reward). During all reward trials, the new reward was delivered at the same location and in the same amount of the reward used during training (familiar reward; Fig. 1C4). Three experimental protocols were designed and termed group I, II and III (Fig. 1C5a–c). Each protocol started with the recording of neuronal activity during navigation of the familiar corridor (Fig. 1C1); these trials are indicated as baseline. Following baseline, mice were teleported to one specific novel corridor (Fig. 1C1–4). In group I experiments (*N* = 3 mice), for 40 consecutive trials, we examined the effects of unexpected visual stimuli on dCA1 firing (Fig. 1C5a). Novel visual stimuli are indicated as cues or novel cues. In group II experiments (*N* = 3 mice) all the novel stimuli were examined, and novel contexts were experienced in alternation to familiar ones (Fig. 1C5b). Each stimulus was presented one time per trial, and repeated in consecutive trials. Odor was tested first, followed by novel rewards and cues. Odor delivery (Fig. 1C3) typically occurred for 20 trials given that, by repeated exposure, olfactory stimuli induce inurement/habituation. A novel reward was employed for 10 trials (NewRew; Fig. 1C4). We also investigated whether dCA1 neurons might detect changes in the significance/value of the rewarding experiences or signal the violation of reward prediction. The effects of an unexpected extra amount of rewards were assessed by employing 3× the amount of the new reward (total volume, ~ 10 µl; ExRew = extra reward) for three consecutive trials (Fig. 1C5b and c, bottom row). Reward omission was evaluated during five trials (Fig. 1C5b and c, bottom row). Ultimately, novel cues were tested as described for group I (Fig. 1C2). Five mice were used for group III experiments (Fig. 1C5c). The corridors employed were similar to group II (Fig. 1C1–4) and the interrogation of unexpected stimuli was conducted as described above. In contrast, the order of stimulus-presentation was inverted and counterbalanced with respect to group II, here consisting of cue novelty first, followed by reward and odor stimuli (Fig. 1C5c).

### Acute tetrode recordings from neuronal populations of the hippocampus dCA1

Acute silicon-probe recordings were performed as previously described (Lasztoczi and Klausberger 2016), with modifications. One probe in 8- or 4-tetrode configuration (Neuronexus) was inserted perpendicular to dCA1, along with the anterior-posterior axis (Figure D1). We aimed at the proximal axis of the pyramidal layer, as this area receives inputs from the lateral EC and is engaged in the computation of olfactory stimuli (Igarashi et al. 2014). To increase stability, we employed a softened wax (50% cacao butter and 50% wax; Sigma). Signals of the dCA1 local field potential (LFP) were pre-amplified with an acute headstage (Tucker-Davies Technologies), then amplified 1000× and low-pass filtered at 6-kHz (Lynx-8 signal conditioners; NeuraLynx). The output signal was fed into an analog-to-digital converter (Power1401; Cambridge Electronic Design) and continuously digitized (2-kHz, for units; ~16.8-kHz up-sampled offline to 20-kHz with a customized MATLAB script). The pyramidal layer was identified by detection of complex spikes and by specific hallmarks of the LFP, such as the amplitude and phase of the theta oscillations (Fig. 1D2). Thus, depending upon probe-location, signals from the neighboring strata were also recorded. Within the virtual corridor, subject positions were acquired at 20-Hz through the Phenosys software and synchronized to the electrophysiological signal via Spike2 (version 7, Cambridge Electronic Design). At the end of each experiment, mice were injected with an overdose of urethane and transcardially perfused with cold saline followed by fixative (4% paraformaldehyde, 0.05% glutaraldehyde, 15% saturated picric acid). The brains were removed and stored overnight at 4 °C in fixative. Brains were cut in 70-µm sections and washed in phosphate buffer. Following incubation with streptavidin conjugated to Alexa488 (Invitrogen, 1:1000; 4 h at room temperature), sections were mounted on slides. Tracks of the silicon probes (Fig. 1E1) were visualized under an Olympus BX61 epifluorescence microscope. To facilitate post-hoc identification of the implantation site, in few experiments the probe was pre-immerged in DiI (Invitrogen; Lasztoczi and Klausberger 2016).

### Spike sorting and data analysis

Units were clustered offline (Csicsvari et al. 2003; Royer et al. 2012). Briefly, spikes with amplitude > 5 standard deviations (SD) were extracted from 0.2-ms windows of the root mean square LFP signal (0.8–5 kHz; Csicsvari et al. 1998). Spike waveforms were sorted into clusters of putative units by an automatic algorithm (KlustaKwik; Harris et al. 2003), and assigned to individual neurons on the basis of their vector clouds, auto-correlograms, and by employing both manual and automatic refinements (Klusters software; Hazan et al. 2006). Our recordings yielded to a total of 192 units. Units were classified into either putative interneurons (32 cells) or putative principal cells on the basis of three parameters, and in accordance with standard criteria assessed during baseline Bs1, i.e.: (1) firing rate; (2) time from first peak to through of the action potentials; (3) burst firing activity/complex spikes. Thus, 32 putative place cells were distinguished from pyramidal non-place cells on the basis of criteria described below (Fig. 1E2, 3); in this study, pyramidal non-place cells are simply indicated pyramidal cells. The classification of units recorded with tetrodes is generally not conclusive and should be considered indicative. Nevertheless, we here refer to interneurons meaning putative interneurons; likewise for pyramidal and place cells.

The impact of novel environments was interrogated in terms of effects on both behavior and neuronal activity and examined during consecutive trials. Although some variability was observed, mice engaged in exploratory behavior for max. the first 10 novel trials. To evaluate the effects on mouse behavior and maze exploration, a conservative approach was employed in which the last five trials of the pre-stimulus baseline were compared to the first five novelty trials (as in Figs. 3A1, 6A1). Space traveled was defined as the space covered from the starting position to the rewarding area; data were then averaged across trials and subjects. The time to complete one trial corresponded to the interval between teleportation and pump activation. Traveling velocity was calculated accordingly. The effects on neuronal connectivity and dynamics were also assessed during given intervals of trials, which we termed blocks. Three-time points were examined. The first block consisted of 10 baseline trials (b-Block), the second block consisted of the first 10 trials in the novel corridor, whereas the remaining (from 11th to the last) novelty trials constituted the third block. Cross-correlograms were employed to determine neuronal interactions and were computed by electing one neuron as a reference and a second as the target cell. Spikes of the reference neuron were binned to the spikes of the target neuron if occurred within a 30-ms window and separated into bins of 1-ms. The zero-point was defined as the time of the reference cell discharge. Interactions between pairs of cells were accepted as significant if a peak occurred within the selected time window, and the peak was of 3-SD above (for excitatory interactions) or below (for inhibitory ones) the mean firing. The effects of novelty on neuron dynamics were examined by computing firing maps. For place cells, the corridor was divided into 4 × 50 bins; rates were calculated by dividing the number of spikes measured in each bin by the time of bin occupancy. The obtained maps were then smoothed with a two-dimensional Gaussian filter. Bins in which the rates were > 20% of the maximal firing were assumed to belong to the place fields (Ciocchi et al. 2015). The stability score was calculated as previously described (Ciocchi et al. 2015). Original fields were obtained during baseline and compared bin by bin with linear correlations to those recorded in novel corridors. Neurons with stability score > 0.37 were considered stable place cells. Space normalized maps were constructed for pyramidal cells and interneurons. The corridor was divided into 50 equally sized bins along its longitudinal axis, and neuronal firing within each bin was computed. Rates were calculated by dividing the numbers of spikes contained within each bin by the time of occupancy across the bin. Firing along the corridor was plotted for each trial. The magnitudes of rate variations were calculated as percent changes from baseline. To assess whether novelty-evoked place cell dynamics exhibited specific space-patterns, the percent of sectors with significant rate variations was determined. Firing was compared among three sectors of the novel corridor, namely: (1) the sector in which the place fields were originally identified (s-PlF, sector of the original place field), (2) sector s1, first sector of compartment c1, which maintained the features of the familiar corridor (s1-Fam; Fig. 1C2-5), and (3) sector s3, first sector of compartment c2, in which novel cue and odor stimuli were first experienced (s3-Nov; Fig. 1C2-5). For cells that did not exhibit a space-restricted firing, average rates of compartment c1 and c2 were obtained from space-normalized maps and compared by employing the 95% Confidence Interval test (95%CI). During baseline trials, two different firing profiles were identified, thus, cells were distinguished into two populations. Neurons for which the firing in c1 resulted within the 95%CI of c2, were classified as c1 = c2. Conversely, if the firing in c1 resulted different from c2, cells were termed c1 ≠ c2. Dynamics during navigation of the novel corridors were evaluated by employing a similar approach. Novelty-evoked firing variations might either occur (1) with the same extent in both compartments or (2) prominently in one of the two compartments. In the former case, neurons of the c1 = c2 and the c1 ≠ c2 groups would maintain the firing profile recorded during baseline and manifest comparable—or uniform—variations between the two compartments. They are termed ‘compartment-Uniform’ (c-Uniform). Conversely, changes occurring preferentially in one compartment would cause the loss of baseline profile. Cells classified as c1 = c2 would turn into cells with the profile of c1 ≠ c2 and the c1 ≠ c2 into c1 = c2. Neurons exhibiting this pattern will be indicated ‘compartment-discriminators’ (c-Discriminators). To determine the effects of novelty with respect to time, we first identified the blocks at which rate variations started (s-Block). Two populations were distinguished. The first population consisted of neurons for which firing during the first 10 novelty trials was different from baseline; in other words, novelty-elicited variations occurred within the first 10 trials. The second population consisted of neurons in which baseline firing resulted comparable to the first novelty block, but differed from the subsequent blocks of 10 trials (block 2, or 3, etc.). We concluded that, for these cells, the effects occurred after the 11th trial. Next, we evaluated the response time-course and determined how changes evolved with the increasing number of trials. For some neurons, firing in any given block > s-Block was out of the confidence interval of the antecedent block, plus exhibited the same direction in rate responses. Thus, rate variations advanced from one trial to the next and we termed these changes progressive. Changes were indicated as reaching a plateau when the firing recorded in any given block > s-Block was comparable to the activity of the antecedent blocks. Variations were classified as reaching a peak when firing in any given block > s-Block was out of the confidence interval of the antecedent block plus the direction of firing resulted opposite with respect to the block in which the effects were first assessed.

Event-triggered, short-latency responses were measured during the presentation of each novel stimulus. Neuronal firing was calculated within a 4-s window preceding and 4-s following the triggering stimulus and divided into 40 bins of 200-ms duration. By meaning the time matrixes across trials, the peri-event time histograms were obtained. Passages through the curtain were elected as triggering stimulus during cue trials; likewise, firing during activation of the odor valve and the reward pump was examined during odor and reward trials, respectively. Thus, for each novel protocols, two values of neuronal activity were obtained: (1) dynamics during maze navigation and (2) event-triggered responses. Given the exiguous number of trials, only event-triggered responses were assessed during extra- and no-reward protocols.

In accordance with previous reports (Su et al. 2001; Mizuseki et al. 2012), neurons fire in burst mode if the inter-spike interval (ISI) is less than 6-msec. Spikes were then sorted into burst categories for events containing at least three spikes and whose duration was less than 20-msec (NeuroExplorer software; courtesy of Dr. Wulf Haubensak). Percent of burst firing was defined as the percent of all spikes occurring in burst mode (Valenti et al. 2011). The effects of novelty on the percent of burst firing, ISI and number of spike per events were evaluated.

We also computed dCA1 neuron firing with respect to the local network oscillations. To evaluate dCA1 coupling to a specific phase of theta rhythm, signals of the LFP from either the pyramidal layer or the proximal stratum oriens were selected and processed as previously described (Klausberger et al. 2005; Lapray et al. 2012). The instantaneous theta phase was extracted by linear interpolation with peaks defined as occurring at 180° and troughs at 0° and 360° (Klausberger et al. 2005). Custom-scripts and built-in functions elaborated in Spyke2 and MATLAB were employed (courtesy of Dr. Lasztoczi), with the support of the Wavelet Toolbox and the Circular Statistics Toolbox (Berens 2009). Following the assessment of uniformity through the Rayleigh’s test (*α* = 0.05; Zar 2010), the coupling phase (mean phase angles), the strength of coupling (mean vector lengths), and the measurements of the theta cycles in which neurons exhibited active firing (active cycles) were computed with circular statistics. Neuronal firing during sharp-waves associated ripples (SWR) is typically assessed during interruption of subject behavior or reward consumption. In contrast, head-restricted mice tend to engage in uninterrupted motion, and we also observed this behavior in our experimental set. To evaluate neuronal activity with respect to SWR, we tried to suspend mouse activity by inserting a 3-s break in dark contexts (i.e. shutting down the images on the screens). Unfortunately, mice exhibited active behaviors also during this time window, making the SWR analysis unreliable. As for gamma oscillation, the analysis of firing coupled to this rhythm depends on recordings of the LFP across all dCA1 layers (Lasztoczi and Klausberger 2014, 2016). Given that this approach cannot be achieved with a silicon probe in tetrode configuration, we could not evaluate neuronal activity during gamma oscillation.

### Experimental design and statistical analysis

All values are expressed as mean ± SEM. Statistical analysis was performed either with MATLAB (MathWorks) or with GraphPad Prism 5 (GraphPad), and all values were accepted as significant for *P* ≤ 0.05. A two-way ANOVA was employed to test the effects of novelty on the velocity of mice navigation, using space (sectors) and conditions (baseline vs. novelty) as factors. The effects of novel stimuli on neuron dynamics and burst firing were assessed by employing the Wilcoxon rank sum test, due to non-normal distribution of the data and assuming that the averaged firing rates are independent between trials. Mann–Whitney test and One-way ANOVA, followed by Bonferroni Multiple Comparison test for post-hoc analysis, were used as indicated in the “Results” section. The effects of novel stimuli on cell coupling to the phases of theta oscillation were evaluated by employing the Wilcoxon rank test analysis or a permutation test (*n* = 60,000). Events triggers short-latency responses were evaluated by employing the paired *t* test.

## Results

### Encountering of unexpected stimuli in a familiar context altered neuronal firing and network connectivity

Previous studies in humans have reported maximal levels of dCA1 activation during tasks where unexpected enclosures are embedded into familiar contexts (Jenkins et al. 2004; Lisman and Grace 2005; Kumaran and Maguire 2006a, 2007a, b, 2009; Eichenbaum et al. 2007; Poppenk et al. 2010; Duncan et al. 2012). Nevertheless, dCA1 firing during encounters of unexpected events, experienced in sequence and within the same context, remains elusive. Likewise, we still do not know whether different stimuli trigger congruent responses or, in contrast, dCA1 neurons can discriminate among events of different nature. To address these questions, we employed acute silicon probe recordings from head-fixed mice navigating virtual environments (Fig. 1). Three novel stimuli were examined: visual cues, an odor perceived as neutral by the mice, and an unexpected/appetible reward. To assess the impact of expectation in novelty processing, novel stimuli were delivered in compartment c2 (Fig. 1C2–4), in environments preserving familiar features. This configuration shall promote a continuous comparison between the familiar and novel compartment, and trigger match/mismatch computation.

**Fig. 2:**
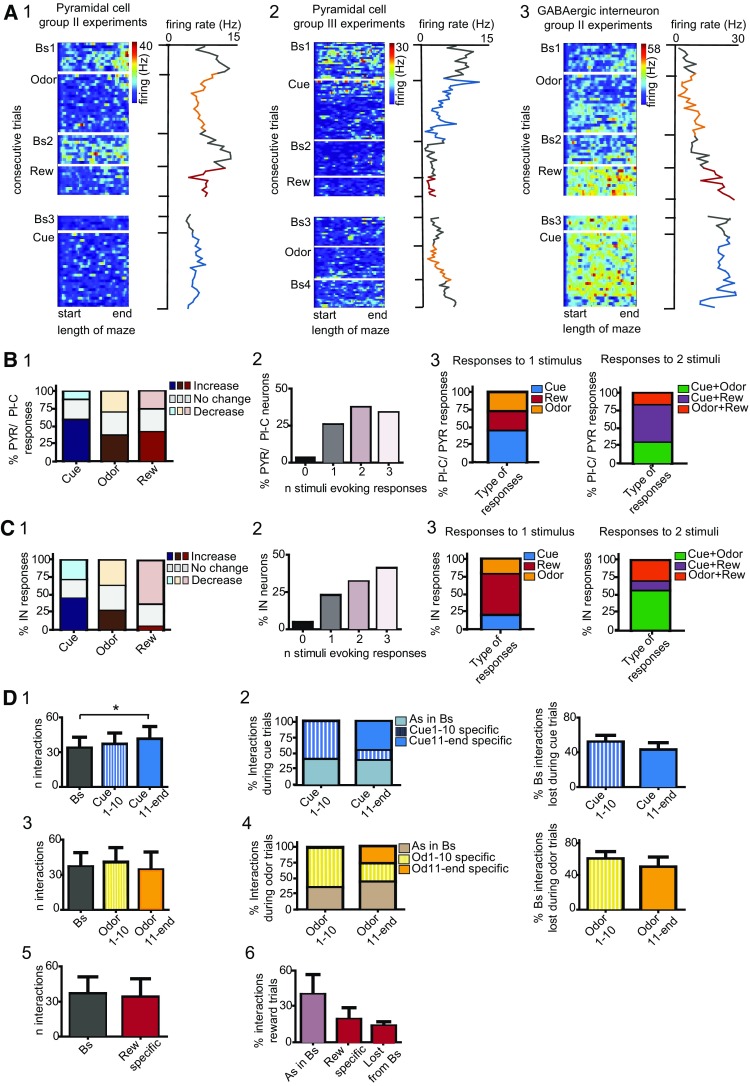
Dorsal CA1 neuronal responses following the experience of novel stimuli. **A** 1–3 Space-normalized map (left) and firing rate histogram (right) for three different neurons during the experience of novel stimuli. 1 exposure to odor induced a rapid decrease in rates that reverted to baseline at the cessation of the stimulus; 3 the experience of a novel reward induced a specific increase in firing. Each row is representative of normalized cell firing during consequent trials. The transition between baselines and novel protocols are indicated by white horizontal bars. Rate values are color-coded, with red color indicating maximal firing; values of max. firing are reported on the right, in Hz. **B** 1 Percent of principal cells exhibiting altered firing in response to the different novel stimuli; the experience of unexpected events triggered opponent directions in firing, as indicated by different tone colors. 2 Neuron susceptibility to the occurrence of the different unexpected stimuli; 71% of principal cells exhibited significant responses to more than one stimulus. 3 Left-Distribution of responses to the three novel stimuli for neurons exhibiting only one significant response. Right-Similar to left, but for cells responding to two stimuli. **C** As in **B**, but for interneurons. **D** For cue trials. 1 Number of neuronal interactions during baseline (Bs), first 10 novel trials (Cue1–10), from the 11th trials to the end (Cue11-end) (One-way ANOVA). 2 Left- Distribution of different types of the recorded interactions; As in Bs = interactions retained from baseline, Cue1–10 specific = interactions elicited during the first 10 trials, Cue11-end specific = interactions established from trial 11. Right, number of baseline interactions lost during the two-time points of the cue-protocol. 3 to 4 As in 1–2, but here for odor trials (One-way ANOVA). 5 to 6 As in 1–3, but for reward trials. Rew Specific = interactions specifically elicited during new reward trials. Significance levels are given as *p < 0.05, **p < 0.01, ***p < 0.001, ****p < 0.0001 throughout the figures. *N* = 8 mice. Principal cells, *n* = 115; interneurons, *n* = 26

To assess whether a pattern could be identified in the responses to different stimuli, we interrogated neuronal firing during group II and III protocols (Fig. 1C5; *N* = 8 mice). The experience of unexpected events in compartment c2 elicited pronounced and stimulus-specific rate variations (Fig. 2A1–3; Suppl 1). We found that 71.1% of principal cells, including also place cells, altered their firing during navigation in the novel cue corridor (Wilcoxon rank sum test; Fig. 2B1). Likewise, novel odor and reward evoked significant rate variations in 67.8 and 65.6% of neurons, respectively (Fig. 2B1). Neuronal responses were further distinguished on the basis of the direction of the elicited dynamics (Figure Suppl. 1). Following exposure to unexpected cues, the majority of principal cells increased their activity; conversely, only in a small percent of neurons decreased rates were measured (Fig. 2B1; Suppl. 1A1). During odor and novel reward trials, a similar fraction of cells manifested both increased and decreased firing (Fig. 2B1; Suppl. 1A2–3). As next, we inquired whether a pattern could be recognized in dCA1 responses to different novel stimuli. To this end, we first assessed whether dCA1 neurons manifest a generalized susceptibility to unexpected stimuli. This should be reflected in random, coherent, and indiscriminate rate variations during the encounters of any novel event. We regarded neurons as having generalized and homogeneous responses if all novel stimuli evoked significant responses, and these responses displayed the same direction. On the other hand, neurons were classified as having heterogeneous responses when at least one of the responses to novel stimuli was different, i.e. either the cells did not respond or the direction of one response was antithetical. We found that only 10% of principal cells exhibited homogeneous responses. For the majority of neurons, the direction of the evoked responses was incongruent; therefore, we classified the novelty-evoked effects as heterogeneous. Noticeably, heterogeneity in neuronal responses would rule out the possibility that the observed effects arise from navigation and general engagement in stereotyped behaviors, or from drifting of the recorded signal (Fig. 2a). Indeed, in these cases, we would expect a continuum in rate variations across the different trials and novel protocols. Rather, this finding indicates that dCA1 neurons exhibited specific changes during the experience of each stimulus, a pattern that might account for their ability to discriminate among different types of events. The majority of principal cells displayed at least one significant response during the encounter of unexpected events (Fig. 2B2). Neurons susceptible to only one stimulus manifested a comparable distribution in the responses to each novel stimulus (Fig. 2B3, left). To assess whether dCA1 principal cells exhibit associated responses during the experience of different novel stimuli, we examined neurons exhibiting two significant responses. Given that both visual cue and odor are sensory stimuli, we assessed whether principal cells responding to odor might also manifest significant responses during the encounters of novel cues. On the other hand, neurons responding to odor might rather exhibit the second response during the experience of novel rewards, as both stimuli trigger associative learning. We found that responses to cue and reward occurred with a higher probability (Fig. 2B3, right). Exposure to novel visual stimuli induced rate changes in 72.7% of interneurons. Interneurons resulted reactive also to the unexpected encounters of odor or novel reward, as we recorded significant effects in 68.2 and 63.6% cells, respectively (Fig. 2C1). When we examined the direction of firing, we found a proportionally similar percent of neurons exhibiting increased or decreased rates. In contrast, the experience of a novel reward typically depressed neuronal activity (Fig. 2C1). Interneurons also displayed heterogeneous responses, with only 18.2% embracing homogeneous activity. In other words, these cells discriminated among different types of stimuli by suitably changing their dynamics at the encounter of each event. Noticeably, 41% neurons exhibited significant rate variations during navigation in all three novel contexts (Fig. 2C2). Among cells responding to only one stimulus, the number of interneurons susceptible to new rewards was slightly higher compared to either cue or odor responses (Fig. 2C3, left). Next, we interrogated whether dCA1 responses might manifest a defined pattern or a specific stimulus-association. The combinatorial responses to both sensory stimuli (visual and olfactory) prevailed (Fig. 2C3, right). Conversely, 28.6% of cells exhibited significant responses to odor and reward, and only 14.3% interneurons signaled the occurrence of cues and rewards (Fig. 2C3, right).

**Fig. 3:**
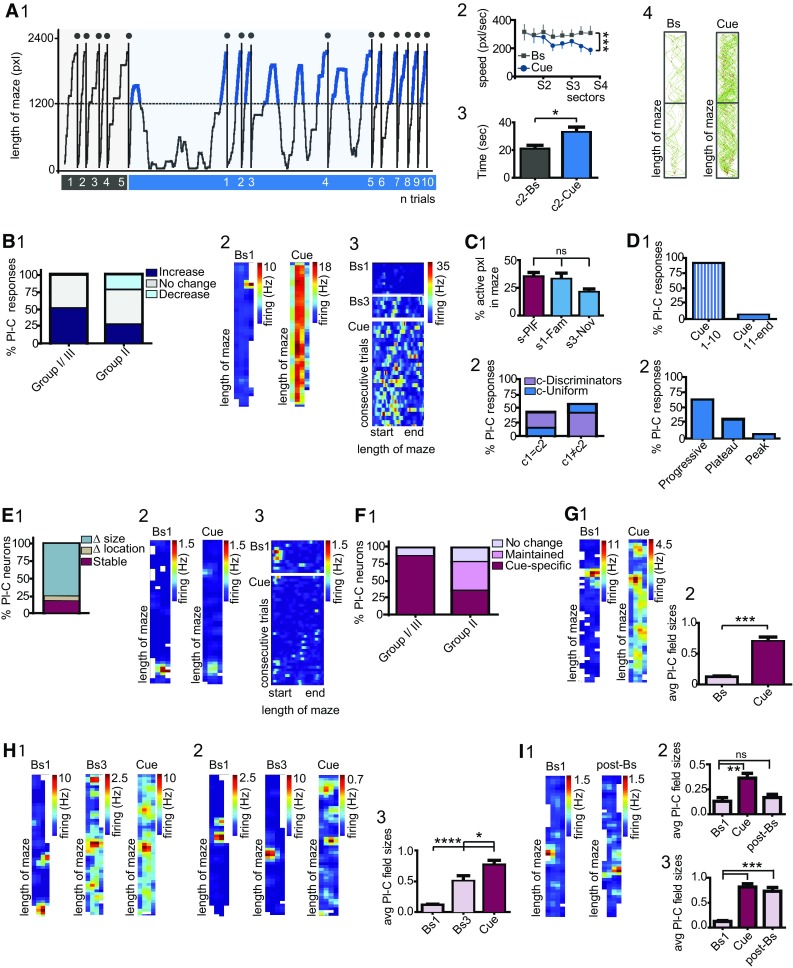
Hippocampal place cells responses during the encountering of novel visual cues. **A** 1 Mice trajectories during maze navigation; rectangles indicate trials examined for behavioral analysis. *X*-axis, numbers indicate trials during baseline (grey bar) and cue (blue bar) protocols. *Y*-axis, space traveled during each trial from starting position (*y* = 0) to reward location (*y* = 2400 pxl); dotted line indicates the curtain. Grey circles (top) = familiar reward. Thick blue lines indicate trajectories in compartment c2, where novel cues were experienced. 2 Specific decrease in mice velocity during navigation in compartment c2 (Two-way RM ANOVA). 3 Time to complete each trial during baseline and cue trials. 4 Maze occupancy and space-associated firing of one neuron. **B** 1 Typologies of responses elicited by cue exposure, expressed in percent of group I/III and II place cells. 2 Firing pattern of one group-I place cell; exposure to novel cues induced loss of the defined place field and increased firing along the entire corridor. 3 Space normalized map for one group-II place cell illustrates cue-elicited dynamics. **C** 1 Distribution of group I/III neuron firing among specific sectors of the cue corridor; *s-PlF* sectors in which the original fields were recorded, *s1-Fam* sector s1 of the familiar compartment, *s3-Nov* sector s3 of the novel compartment. 2 Distribution of firing for group-II cells during cue trials, rate variations were not associated to a specific location; see Experimental procedures. **D** 1 Onsets of place cell responses to novel cues; 2 Means by which rate variations advanced during consequent trials. **E** 1 Typologies of the effects evoked by novel cues on place fields; Δ sizes = increased sizes, Δ location = transitory changes in field locations, stable = stable fields. 2 Place maps recorded for one neuron in which exposure to novel cues induced a transitory delocalization of the field. 3 normalized map of the same neuron. **F** 1 Distribution of the different effects on field stability for group I/III and II neurons; no change = stable fields, maintained = changes in field size were induced by previous stimuli, and then maintained during cue trials, cue-specific = cue-evoked specific increase in sizes. **G** 1 Increased field size and delocalized pattern of firing for one group I place cell; 2 quantification of the size increases for group I/III cell place cells (Mann–Whitney test). **H** Firing maps for two group II neurons; (1) exposure to cue-novelty induced an additional increase in sizes; (2) cue-specific delocalization and enlargement of the place field. 3 Quantification of size increases for group II neurons (One-way ANOVA, *p* < 0.05). **F** 1 Firing maps for one place cell exhibiting a return of field sizes within baseline. 2 Quantification of field sizes measured during pre- and post-Bs for neurons in which the sizes reverted toward pre-Bs values. 3 as in 2 but for cells in which post-Bs field resulted different (One-way ANOVA). Group I/III, *N* = 8 mice. *n* = 18 cells. Group II, *N* = 3 mice; *n* = 14 cells

Previous studies indicate that, during cognitive tasks, place cell computation is supported by changes in interneuron dynamics that follow the flickering of new assembly formation or the fade of old ones (Fyhn et al. 2002; Dupret et al. 2010, 2013). Accordingly, we would expect that during the encoding of unexpected contexts a rearrangement in local networks occurs, which seemingly should be reflected in altered neuronal interactions (see Experimental procedures). The encounter of unexpected cues elicited an increment of dCA1 interactions, especially during the second block of novel trials (Bs = 33.5 ± 9.4, Cue1–10 = 37 ± 9.6, Cue11-end = 41.4 ± 10.8; Bs vs. Cue11-end, *F*(2, 20) = 4.902, *p* = 0.018, One-way RM ANOVA; Fig. 2D1). Although during the first 10 trials no significant differences in the actual numbers of interactions were observed, we found some differences in the nature of the connections. During Cue1-10, a certain number of baseline interactions were retained, whereas stimulus-specific interactions emerged (Fig. 2D2, left). Cue11-end trials also consisted of a mixture of interactions. Thus, we identified both interactions retained/retrieved from either baseline and/or Cue1-10 trials, as well as new interactions emerging during the last trials of the cue-protocol (Fig. 2D2, left). Conversely, both cue-blocks were characterized by the loss of a significant number of interactions with respect to baseline (for Cue1–10) or baseline and Cue1-10 (for Cue11-end; Fig. 2D2, right). For odor trials, no differences in the number of interactions were measured when the three-time points were compared (Bs, Odor1-10, Odor11-end; *p* = 0.699, One-way RM ANOVA; Fig. 2D3). Nevertheless, the experience of an olfactory stimulus evoked a rearrangement in network connectivity (Fig. 2D3, 4), which resulted in new interactions established during both the first 10 trials as well as the subsequent odor trials (Odor1-10 and 11-end specific; Fig. 2D4, left). Similar to cue trials, we also observed that ~ 1/3 of baseline interactions were retained during navigation of the odor corridor (Fig. 2D4, left), whereas others were lost (Fig. 2D4, right). The experience of a novel reward induced no significant changes in the actual number of interactions (Bs vs. Rew; *p* = 0.798, Mann–Whitney test; Fig. 2D5), although we measured a similar reshuffling in connectivity as described for the other two novel stimuli (Fig. 2D6).

### Hippocampal dCA1 neuron dynamics during the experience of novel visual stimuli

Exposure to environments different in either their geometry or in sensory stimuli elicits global- or rate place cell remapping, respectively, in hippocampal place cells (Muller and Kubie 1987; Bostock et al. 1991; Anderson and Jeffery 2003; Leutgeb et al. 2005). Learning and the encoding of novel environments are thought to be initiated by a failure in expectation, although the actual site of novelty detection is currently a matter of debate. Moreover, data on dCA1 responses during the encoding of unexpected events, embedded in otherwise familiar environments, are puzzling. To address these questions, we investigated dCA1 neuron firing during the encounter of unexpected visual stimuli, which were delivered in otherwise familiar environments. We also aimed at elucidating whether this computation might be affected by an increasing number of performed trials, which imply response adaptation to a context no longer unexpected. We first assessed whether the exposure to a novel context (Fig. 1C5) might be reflected in alterations of mice performance and behavior. While navigating a familiar corridor (Fig. 1C1), mice covered an average space of 2661 ± 324.9pxls; the average time to complete each trial resulted of 20.6 ± 2.8 s and mice traveled the corridor with a speed of 265.8 ± 31.11pxl/s. Teleportation to the novel cue corridor (Fig. 1C2) elicited an increase in exploratory behavior. Mice tended to accomplish multiple passages before accessing the rewarding area, during which they also increased the approaches to the walls displaying novel cues (Fig. 3A1). The increment in maze travels was associated to a decrease in velocity within the novel compartment c2 (*F*(1, 80) = 27.42, *p* < 0.0001, Two-way Repeated Measure ANOVA; Fig. 3A2). In addition, a significant increase in the time to complete each trial was measured (Cue trials, 32.6 ± 4.0sec; *U* = 29.00, *n*1 = *n*2 = 11, *p* = 0.021, Mann–Whitney test, Gaussian distribution; Fig. 3A3). The percent of maze occupied during navigation was of 79.0 ± 4.3% for the familiar and 95.5 ± 1.5% for the cue corridor (Fig. 3A4).

Next, we sought to evaluate the impact of unexpected visual cues on dCA1 place cell dynamics (see Experimental procedures). Given that for group I (*N* = 3 mice) and III experiments (*N* = 5) novel stimuli were presented under similar conditions (Fig. 1C5), data were combined. We found that exposure to novel cues significantly increased the rates of 50% neurons (Wilcoxon rank sum test; Fig. 3B1). This effect consisted in a dramatic loss of the characteristic place cell-restricted firing, which unfolded toward an undefined pattern of activity along the corridor (Fig. 3B2). The experience of novel visual stimuli occurred after odor and reward for group II neurons (*N* = 3 mice). As mentioned above, these stimuli also promoted significant rate variations. Consequently, the place fields recorded during pre-cue baseline were not definite, rather patches of activity were detected in several spots of the corridor, and these variations were often carried toward pre-cue baselines (Fig. 3B3). Navigation of the cue corridor induced additional changes in 50% of group II neurons (Wilcoxon rank sum test; Fig. 3B1, 3), indicating that novel cues elicited specific responses even when occurring within the context in which other novel events were experienced. Both enhanced (28.6% cells; Fig. 3B1, 3) and reduced dynamics (21.4% cells; Fig. 3B1) were measured. In group II, the magnitudes of the decreased responses were quantified as 50.6 ± 8.0% change from baseline. When the magnitudes of the group II and III rate increases were compared, no significant difference was detected, resulting of 313.5 ± 144.8% change (*p* = 0.148, Mann–Whitney test). Given that novel cues were presented in compartment c2, we interrogated whether rate variations might be preferentially elicited within the novel compartment. For group I/III neurons, we compared firing in three sectors of the cue corridor (see Experimental procedures). The dynamics elicited by the encounter of novel cues manifested no specific space-association and changes occurred with similar probabilities in all three sectors (*p* = 0.065, One-way ANOVA; Fig. 3C1). For 72.2% cells, elicited-dynamics developed along all four sectors (see Fig. 3B2). With respect to group II, 42.8% of cells were identified as c1 = c2 neurons during navigation of the familiar corridor (95%CI). Exposure to novel cues induced a prominent reorganization in firing, resulting in 28.6% of these cells exhibiting variations associated to one of the two compartments (c-Discriminators; Fig. 3C2). Conversely, 42.8% of c1 ≠ c2 cells lost their profile and manifested similar dynamics between the two compartments (Fig. 3C2). Despite these rearrangements, cue-evoked variations did not specifically occur within the novel compartment. Analysis of behavior indicated that variations in mice’s performance occurred in coincidence to teleportation to the cue corridor (Fig. 3A1). To evaluate how dCA1 neuron responses evolve during repeated exposure to novel cues, we determined the onset as well as the time course of the cue-elicited responses. The experience of unexpected cues induced changes in firing that, for 93.8% place cells, occurred within the first 10 trials (95%CI; Fig. 3D1). With the increasing number of performed trials, different patterns of responses were identified. Thus, 62.5% of place cells exhibited rate variations that, once initiated, continued for all remaining trials and we termed this effect progressive (95%CI; Fig. 3D2). For 31.2% cells, the evoked changes reached a plateau, whereas, in a smaller percentage of cells, a peak value, to then invert the direction towards baseline firing (Fig. 3D2). Following a defined number of trials, mice were teleported to the familiar corridor, and the average rates recorded during pre-cue (pre-Bs) and post-cue baselines (post-Bs) were compared. For 72.3% of place cells, pre-Bs firing resulted significantly different from post-Bs (Bs1 = 0.64 ± 0.2 Hz; Bs2 = 6.8 ± 3.1 Hz; U = 2.0, *n*1 = *n*2 = 6, *p* = 0.0043, Mann–Whitney test).

To elucidate whether place cells that did not exhibit altered firing dynamics might signal the occurrence of novel cues by remapping (Muller and Kubie 1987), we examined the stability of the place fields. We assessed a significant loss in stability for 81.2% of neurons (Fig. 3E1). For two neurons, the place fields disappeared from the original location; nevertheless, these changes were undefined and transitory (Fig. 3E2, 3) indicating that the encounter of unexpected visual stimuli did not induce global remapping. In contrast, for the majority of neurons, this loss consisted of increased field sizes (Fig. 3E1). The magnitude of this increase was quantified within each experimental group. In 88.9% of group I/III neurons, navigation of the cue-corridor elicited a significant increase of the field sizes (Bs = 0.1 ± 0.01, Cue = 0.7 ± 0.07; *U* = 0.000, *n*1 = *n*2 = 15, *p* < 0.0001, Mann–Whitney test; Fig. 3F1, G1-2) and, for 38.9% of cells, the increments occurred without affecting cell firing. For the majority of group II neurons, previous exposure to odor and reward promoted a loss of the place-associated firing (Fig. 2; also see analysis below), which was also observed during Bs3. Nevertheless, the encounter of novel cues evoked additional changes (Bs1 = 0.1 ± 0.02, Bs3 = 0.5 ± 0.09, Cue = 0.85 ± 0.06; *F*(2, 33) = 21.13, *p* < 0.0001, One-way ANOVA; Fig. 3F1, H1–3). Thus, while 42.9% of neurons retained the increase elicited by other novel events (Fig. 3F1, H1), for 35.7% of group II neurons the encountered of unexpected cues evoked stimulus-specific responses (Fig. 3F1, 3H2). To assess whether a return to the familiar corridor might be associated with re-sizing of the fields, we compared Bs1 to post-Bs. Of all place cells recorded, 76.9% maintained the cue-evoked increase in field sizes (Bs1 = 0.1 ± 0.02, Cue = 0.8 ± 0.06, post-Bs = 0.7 ± 0.07; Bs1 vs. post-Bs, *F*(2, 33) = 39.52, *p* < 0.0001, One-Way ANOVA; Cue vs. post-Bs, One-Way ANOVA, *p* > 0.05; Fig. 3I1, 3).

**Fig. 4:**
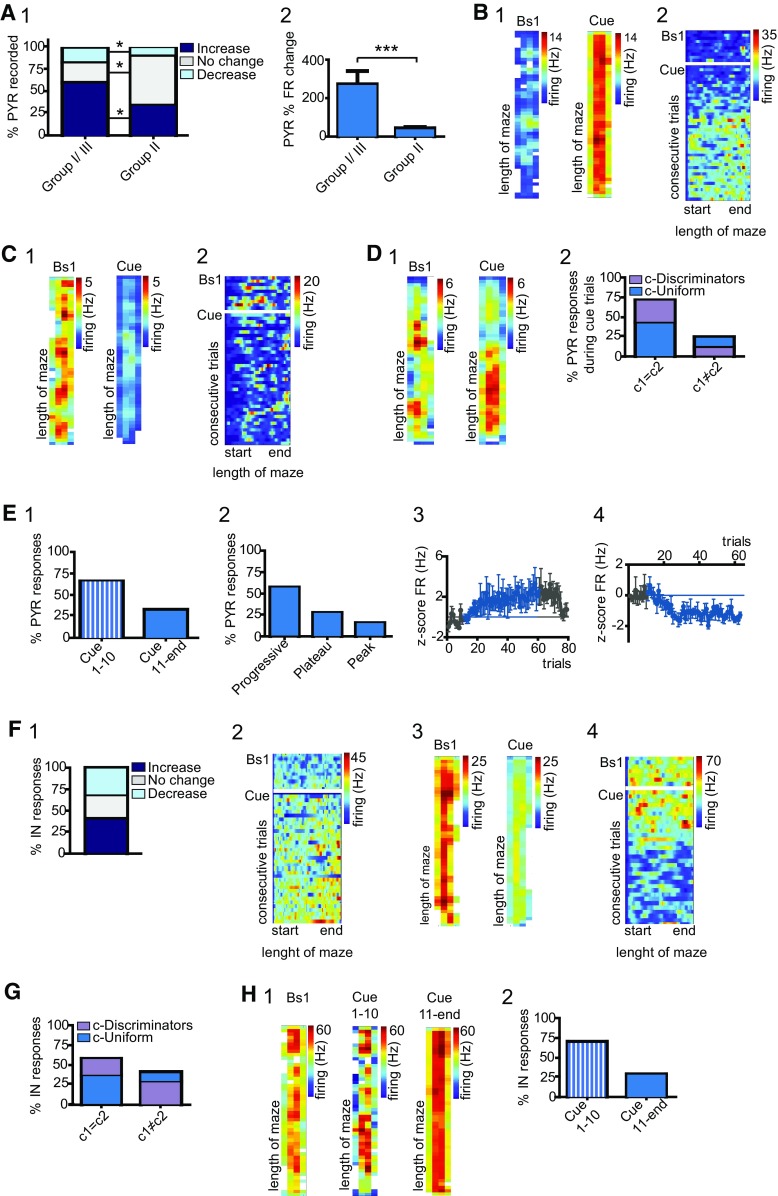
Pyramidal cell and interneuron responses to novel visual stimuli. **A** 1 Percent of group I/III and group II pyramidal cell responses during cue trials; note the significant difference in response distribution (95% CI). 2 Magnitudes of the cue-evoked increase for group I/III and group II neurons (Mann–Whitney test). **B** 1 Cue-evoked rate increase for one group I/III pyramidal cell. 2 Delayed onset of the novelty evoked rate variations for a different group I/III neuron. **C** 1 Cue-induced decreased rate for another group I/III cell. 2 Rapid onset of rate variations for a different neuron. **D** 1 For this group II cell, changes in firing occurred within compartment c1. 2 Pattern of the cue-evoked dynamics with respect to the two compartments, the pronounced rearrangement in firing did not exhibit space-selectivity. **E** 1 Distribution of cell responses with respect to the onset of the evoked changes. 2 Once elicited, variations advanced according to different firing pattern. 3 Example of progressive rate variations elicited by the cue-protocol, changes continued for all novelty trials and reverted to pre-Bs during recordings of post-Bs. 4 Plateau in cue-evoked rate variations. **F** 1 Percent distribution of interneuron responses during cue trials. All groups combined. 2 For this interneuron, enhanced firing activity occurred primarily in c2 and were delayed with respect to stimulus presentation, 3 Decreased rates during cue trials for a different interneuron. 4 Delayed decrease in dynamics for a different cell. **G** Similar to D2, but here for interneuron responses. **H** 1 For this interneuron, cue-evoked firing variations occurred within the first 10 trials and advanced during the subsequent trials. 2 Distribution of the onset of responses for all interneurons. Group I/III, *N* = 8 mice; *n* = 82 PYR, *n* = 22 IN. Group II: *N* = 3 mice; *n* = 46 PYR; *n* = 10 IN

The long axons of not only place cells but also of pyramidals project to brain structures that are engaged in the processing of unexpected events. Accordingly, changes in pyramidal cell activity might reveal the contribution of these cells in carrying novel information to downstream structures. The experience of novel cues induced rate variations in the majority of group I/III pyramidal cells (Fig. 4A1). In 61.0% of neurons, these changes were assessed as enhanced activity (Wilcoxon rank sum test; Fig. 4A1, 4B), whereas the remaining cells displayed attenuated rates (Fig. 4A1, 4C). Navigation of the cue corridor affected also the activity of 43.5% group II pyramidal cells (Wilcoxon rank sum test; Fig. 4A1). When comparing the responses of group II to group I/III, we recognized a pronounced reduction in the number of both increased and decreased responses (95%CI; Fig. 4A1). The two groups differed also for the magnitude of the evoked responses (*U* = 75.00, *p* < 0.0001, Mann–Whitney test; Fig. 4A2), that for group I/III was quantified as 272.4 ± 68.7%, whereas for group II as 43.5 ± 6.4% change. Taken together, these data indicate that a previous exposure to different novel stimuli alters pyramidal cell susceptibility to novel cues, maybe suggesting a mechanism of stimulus-cross sensitization. To evaluate whether the experience of a novel context might induce changes associated with specific space of the corridor, we compared firing between the two compartments. During navigation of the familiar corridor, pyramidal cell activity resulted fairly distributed in space, with 71.1% of neurons manifesting a c1 = c2 profile. Exposure to novel cues induced a rearrangement in 44.6% cells, leading to a slight increment of neurons exhibiting c-Discriminator profiles (95%CI; Fig. 4D1, 2). Nevertheless, the elicited dynamics were not associated with the novel compartment. For 66.7% of pyramidal cells, cue-evoked dynamics occurred within the first 10 trials (95%CI; Fig. 4E1). Analysis of the time-course of the evoked responses revealed different patterns of activity, with 57.1% of neurons exhibiting progressive changes (95%CI; Fig. 4E2–4). At cessation of the cue protocol, the post-Bs firing of 81.8% cells resulted significantly different in respect to pre-Bs (Fig. 4E1), suggesting that cue-evoked changes were retained also in the absence of the triggering stimulus (pre-Bs = 2.6 ± 0.5 Hz, post-Bs = 6.4 ± 1.2 Hz; *U* = 390.0, *p* = 0.002, Mann–Whitney test).

At last, we examined the effects of novel cues on the activity of GABAergic interneuron. Similar effects were estimated both in terms of percent of responding cells (95%CI) and of the magnitude of group I/III and group II responses (*p* > 0.05, Mann–Whitney test), and data were combined (Fig. 4F1). Novel cues elicited significant firing variations in 40.6% of interneurons (Wilcoxon rank sum test; Fig. 4F1, 2), measured as 43.7 ± 11.7% increase from baseline. Conversely, 31.2% of cells displayed decreased activity (Fig. 4F1, 3–4), quantified as 28.1 ± 3.2% change. We investigated whether these variations might preferentially occur within a specific space or compartment. On the basis of their baseline profile, 58.3% of interneurons were classified as c1 = c2. During cue-protocol, 20.8% of c1 = c2 cells manifested rate variations that prominently occurred within one compartment (95%CI; Fig. 4G). Coincidentally, we also recorded 12.5% of c1 ≠ c2 neurons that lost their discriminatory profile (Fig. 4G). Despite this reorganization, changes were not associated with compartment c2. Rate variations occurred within the first 10 trials for 70.8% interneurons (95%CI; Fig. 4H1, 2). In 45.8% cells, the observed changes resulted progressive and continued for all the examined trials (Fig. 4I1), whereas, in other cells, changes either reached a plateau or a peak value (25 and 29.2% of neurons, respectively). At cessation of the cue-protocol, rates resulted significantly different from pre-Bs only for 50% of interneurons (pre-Bs = 24.2 ± 3.8, post-Bs = 11.4 ± 1.5; *U* = 2.000, *p* = 0.0087, Mann–Whitney test).

**Fig. 5:**
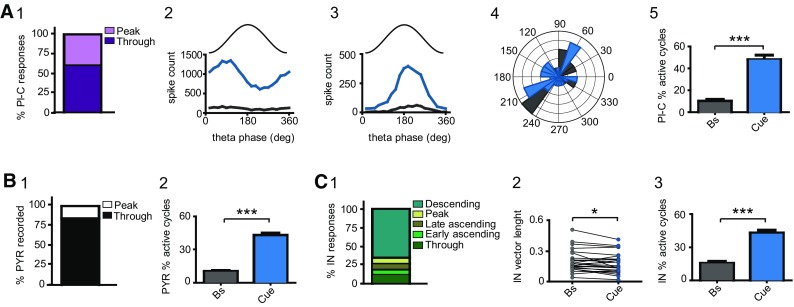
Effects of novel cue experience on the firing coupling to theta oscillation. **A** 1 Distribution of place cell firing with respect to specific phases of local theta oscillation. 2 Firing-coupling for one place cell, which typically fired at the trough of theta during baseline trials (grey trace). Note, exposure to cue novelty (blue) did not affect the coupling phase. 3 Same as in 2, but for one neuron coupled to the peak. 4 Polar plot for all place cells (permutation test). 5 Significant increase in active theta cycles during navigation in the cue corridor (Wilcoxon Rank test). **B** 1 The majority of pyramidal cells were preferentially coupled to the trough of theta oscillation. 2 Cue-evoked increase in active theta cycles (Wilcoxon Rank test). **C** 1 Percent distribution of interneuron firing with respect to specific phases of the theta rhythm. 2 Decreased vector length during navigation in cue corridor (Wilcoxon Rank test). 3 Increase in active cycles following exposure to novel cues (Wilcoxon Rank test). *N* = 8 mice; *n* = 25 PlC; *n* = 90 PYR, *n* = 26 IN

### Effects of novel cues on the coupling to local theta oscillation

Lever and colleagues (2010) reported that, in response to spatial changes, dCA1 neurons shift their firing with respect to theta oscillation and tend to fire closer to the peak, which corresponds to the phase where long-term potentiation has the highest probability to occur. This deviation should ultimately foster learning and the encoding of new experiences (Hasselmo et al. 2002; Hasselmo 2005). To elucidate whether similar changes might occur in our experimental sets, we examined the impact of unexpected visual stimuli on the coupling phase of dCA1 neurons to theta oscillations.

During navigation of the familiar corridor, the firing of 60% place cells was coupled to the trough of local theta (Fig. 5A1). Exposure to novel visual stimuli did not significantly alter either the phase (*p* = 0.972, Permutation test; Fig. 5A2–4) or the strength of coupling (*p* = 0.726, Wilcoxon Rank test, Gaussian approximation). However, we observed a significant increase in active theta cycles (Bs = 10.1 ± 1.7; Cue = 49.0 ± 4.1; *p* < 0.0001, Wilcoxon Rank test, Gaussian approximation; Fig. 5A5). Similar data were collected for pyramidal cells. Baseline recordings revealed that 84.4% of neurons were coupled to the trough of theta (Fig. 5B1). Teleportation to the novel cue corridor increased the number of active theta cycles (Bs = 10.3 ± 0.8; Cue = 43.0 ± 2.0; *p* < 0.0001, Wilcoxon Rank test, Gaussian approximation; Fig. 5B2) without altering either the phase (vector angle; *p* = 0.931, permutation test) or the strength in coupling (vector length; *p* = 0.557, Wilcoxon Rank test, Gaussian approximation).

**Fig. 6:**
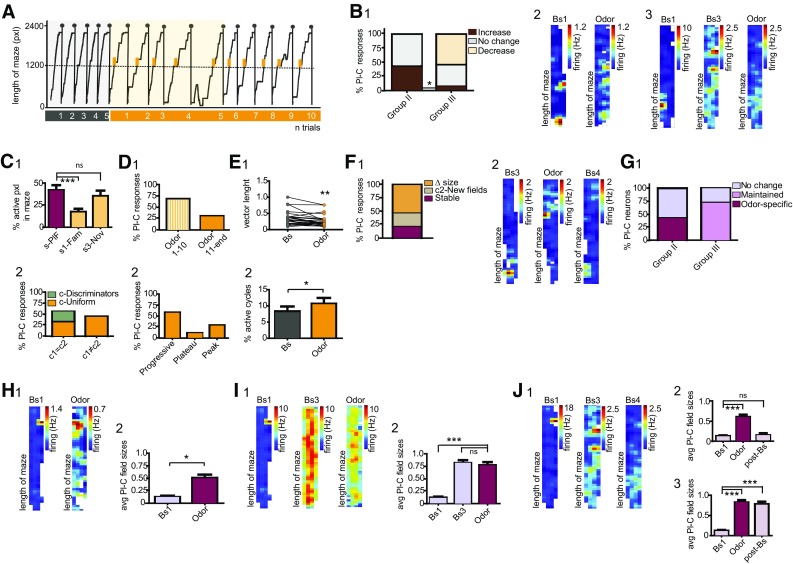
Place cell responses to odor novelty. **A** Trajectories of mice’ laps during odor trials; odor delivery (yellow rectangles) occurred immediately after the crossing of the curtain. **B** 1 Typologies of group II and group III place cell responses evoked by the experience of odor; note the different percent distribution. 2 Odor-elicited indistinct patterns of activity in this group II place cell. 3 Decreased rates during odor trials for one group III neuron. **C** 1 Pattern of space-related firing for group II place cells; odor elicited sector-specific variations in these neurons. 2 Space-related firing for group III place cells. Note, odor altered the baseline firing of these neurons ultimately leading to increased number of cells with similar activity in the two compartments. **D** 1 Onset of odor-elicited responses. 2 Different patterns of dynamics during consequent trials. **E** 1 Odor attenuated the strength of coupling to theta oscillation, and 2 increased the percent of active theta cycles. **F** 1 The experience of odor altered the stability of the place fields. c2-New fields = changes in field location occurring within compartment c2. 2 For this place cell, odor promoted the formation of one novel field in c2. **G** 1 Distribution of the effects on the field size. **H** 1 The experience of odor induced field increase in this group II cell; 2 magnitude of the odor-evoked increase in group II neurons (Mann–Whitney test). **I** 1 In this group III neuron, the experience of odor did not affect field size; 2, magnitudes of the evoked changes in field sizes for group III neurons (One-way ANOVA). Note, field increases occurred during previous stimuli and were then maintained. **J** 1 For this place cell, the field recorded during post-Bs resulted different from pre-Bs. 2 Magnitude of field sizes for place cells exhibiting different fields between pre- and post-Bs; 3 field magnitudes for cells exhibiting similar sizes. Group II: *N* = 3 mice; *n* = 14 PlC. Group III, *N* = 5 mice; *n* = 16 PlC

During baseline, the activity of the GABAergic interneurons, with respect to the phase of local theta rhythm, was consistent with the profile of the different types of the cells located in stratum oriens, pyramidale and radiatum (Fig. 6C1; Somogyi and Klausberger 2005). Unexpected cues decreased the strength of coupling (vector length: Bs = 0.2 ± 0.02; Cue = 0.18 ± 0.02; *p* = 0.029, Wilcoxon Rank test, Gaussian approximation; Fig. 5C2) and elicited an increase in active cycles (Bs = 15.5 ± 1.7; Cue = 42.9 ± 2.5; *p* < 0.0001, Wilcoxon Rank test, Gaussian approximation; Fig. 5C3). However, no effects on the phase of coupling were observed (*p* = 0.683, Permutation test).

### Variations in dCA1 neuronal activity following the experience of odor in a familiar context

**Fig. 7:**
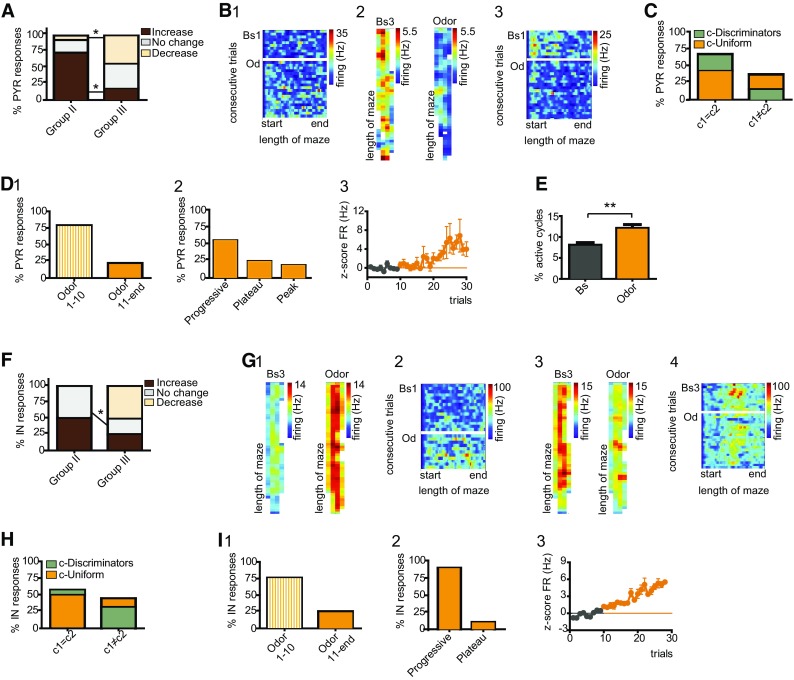
Pyramidal cell and interneuron responses to odor novelty. **A** Percentage of group II and group III pyramidal cell responses during odor trials (95% CI). **B** 1 Normalized map of one pyramidal neuron exhibiting increased firing. 2 The encounter of odor induced decrease activity in this cell. 3 For this neuron, decreased firing started within the first 10 trials. **C** Space-related activity with respect to the two compartments. **D** 1 Onsets of the odor-elicited variations. 2 Variations occurred progressively for the majority of pyramidals, although other patterns of responses were also measured. 3 Changes occurred with a delay in respect to stimulus presentation. **E** Odor increased the percent of active theta cycle in all groups. **F** Distribution of interneuron responses to odor. Note the increment in inhibitory responses for group III cells. **G** 1 The experience of odor, following cue and reward, evoked additional and specific changes in this group III interneuron. 2 Normalized map shows increased dynamics for one group II cell. 3 Example of odor-evoked decreased dynamics for one group III interneurons; 4 as in 2, but here showing decrease dynamics. **H** As in **C**, but for interneuron responses. **I** 1 Onset of the odor-evoked responses in interneurons. 2 Different patterns of responses recorded during the advancement of the task. 3 Time-course for one interneuron showing an immediate and progressive increase in firing. Group II: *N* = 3 mice; *n* = 46 PYR; *n* = 10 IN. Group III, *N* = 5mice; *n* = 44 PYR, *n* = 16 IN

Likewise changes in the spatial properties of the environment, also the experience of odors typically alters hippocampal activity. We assessed whether dCA1 neurons signal the occurrence of an unexpected odor through responses congruent to those evoked by novel cues. Navigation of the odor corridor did not significantly alter mice behavior (Fig. 6a). The time spent to complete each lap (*p* = 0.278, Mann–Whitney test), space traveled (*p* = 0.382, Mann–Whitney test), and the velocity (*p* = 0.324, Two-way RM ANOVA) were not different from the familiar corridors. Maze occupancy resulted in 75.8 ± 3.5% during baseline trials and 89.6 ± 3.5% in odor corridors. The encounter of an unexpected odor, delivered within a familiar context, evoked a significant rate increase for 42.9% of group II neurons (Fig. 6B1), calculated as 227.9 ± 80.2% change from baseline. As reported for cues, these variations consisted in loss of the space-restricted firing and indistinct activity within the maze (Fig. 7B2; Suppl 1B8). When occurred as the last stimulus, odor elicited variations in 54.5% of cells (Wilcoxon rank sum test; Fig. 6B1). However, group III responses mostly consisted in decreased firing (Fig. 6B1, 3), quantified as 35.7 ± 7.8% changes. Next, we investigated whether odor-evoked effects might occur in association with the novel compartment c2. During odor trials, group II neurons tended to fire both within the sectors in which the original fields were recorded (s-PlF) as well as in sector s3 (s-PlF = 41.9 ± 5.6%, s1-Fam = 17.3 ± 3.6%, s3-Nov = 35.2 ± 6.2%; s1-Fam vs. s3-Nov, *F*(2, 39) = 5.826, *p* = 0.0061, One-way ANOVA; Fig. 6B2, 6C1). In other words, although often maintaining a sustained firing within s-PlF, place cells manifested additional patches of activity within the sector in which the odor was experienced. For group III, we recorded 55.6% of neurons that during baseline exhibited a c1 = c2 profile; following teleportation, 22.2% of these neurons developed higher activities within one of the two compartments. Conversely, all c1 ≠ c2 neurons lost their discriminatory activity and developed uniform dynamics along the maze (95%CI; Fig. 6C2). For the majority of place cells, the effects of odor occurred within the first 10 trials (Fig. 6D1), and in 54.6% of neurons, progressed with the increasing number of trials (Fig. 6D2). Finally, for all neurons, the values of post-Bs firing resulted not significantly different from pre-Bs (*p* = 0.694, Mann–Whitney test). Thus, place cells changed their firing at the encounter of the odor and returned to baseline activity when the stimulus was no longer experienced. With respect to theta oscillation, a significant decrease in the strength of coupling was assessed (Bs = 0.3 ± 0.04, Od = 0.3 ± 0.03; *p* = 0.005, Wilcoxon signed rank test; Fig. 6E1) together with increased number of active cycles (Bs = 8.3 ± 1.5, Od, odor = 10.6 ± 1.8; *p* = 0.03, Wilcoxon signed rank test; Fig. 6E2). Exposure to odor did not alter the coupling-phase to the theta rhythm (*p* = 0.951, Permutation test). The encounter of odor affected the stability of 76% place fields (all group combined; Fig. 6F1, 2). For 24% of neurons, the loss of field stability culminated in the coincident formation of new fields, which typically emerged within the novel compartment (Fig. 6F1, 2). For other 52% of place cells, this loss resulted in increased field sizes (Fig. 6F1). We then examined the effects of odor on field sizes. In group II, odor enlarged the fields of 42.9% neurons (Bs1 = 0.1 ± 0.03, Od = 0.5 ± 0.07; *U* = 0.000, *p* = 0.0286, Mann–Whitney test; Fig. 6G1, H1-2). For group III, field sizes recorded during odor trials also resulted significantly different compared to the original fields (Bs1 = 0.1 ± 0.02, Bs3 = 0.8 ± 0.05, Od = 0.8 ± 0.06; Bs1 vs. Bs3, *F*(2, 30) = 58.43, *p* = 0.0041, One-way ANOVA; Fig. 6G1; 6I1-2). Noticeably, however, odor-evoked fields were not different from pre-Bs (Bs3 vs. Od, *p* > 0.05, One-way ANOVA; Fig. 6I1-2). Thus, we concluded that the changes in sizes were not triggered by the odor, rather elicited by the experience of the other stimuli and then carried toward odor trials (Fig. 6H1-2). During post-Bs, 30% of neurons exhibited sizes comparable to the original fields (Bs1 = 0.1 ± 0.02, Od = 0.6 ± 0.06, post-Bs = 0.1 ± 0.05; Bs1 vs. Od, *F*(2, 6) = 31.20, *p* = 0.0007, One-way ANOVA; Od vs. post-Bs, One-way ANOVA, *p* > 0.05; Fig. 6J1-2), indicating that these fields reverted to the original values. However, for the majority of neurons, sizes recorded during pre-Bs resulted different from post-Bs (Bs1 = 0.1 ± 0.02, Od = 0.8 ± 0.06, post-Bs = 0.8 ± 0.06; Bs1 vs. Od, *F*(2, 30) = 58.43, *p* < 0.0001, One-way ANOVA; Fig. 6J1, 3).

Odor presentation enhanced firing states in 73.9% of group II pyramidal cells (Wilcoxon rank sum test; Fig. 7A, B1), whereas other neurons exhibited decreased activities (Fig. 7A). Similar results were found for group III neurons (Fig. 7A, B2, 3). Although the magnitudes of the elicited responses were similar between the groups (Mann–Whitney test; 86.5 ± 15.4% increase and 17.6 ± 2.3% decrease), the distribution of responses resulted different, with a net increment of group III cells exhibiting decreased firing (Fig. 7A). Next, we inquired whether rate variations could manifest space-selectivity. During baseline, 64.9% of neurons were identified as c1 = c2. Exposure to novelty altered the firing distribution of dCA1 pyramidal neurons and, during odor trials, 24.3% of c1 = c2 cells manifested different dynamics between the two compartments (95%CI; Fig. 7C). Conversely, for 21.6% of c1 ≠ c2 neurons, variations resulted similar between the two compartments (Fig. 7C). For 78.1% of pyramidal cells, changes occurred within the first 10 trials (Fig. 7D1) and, for the majority of neurons, progressed with the increasing number of trials (Fig. 7D2, 3). Post-Bs firing was significantly different from pre-Bs activity for 63% of pyramidal cells (pre-Bs = 2.2 ± 0.5, post-Bs = 5.5 ± 1.1; *U* = 343.0, *p* = 0.004, Mann–Whitney test). Analysis of the firing in reference to the local field revealed that odor increased the percent of active theta cycles (Bs = 8.0 ± 0.6, Od = 12.1 ± 0.9; *p* < 0.0001, Wilcoxon signed rank test; Fig. 7E), without affecting either the vector angle (*p* = 0.747, Permutation test) or the vector length (*p* = 0.074, Wilcoxon signed rank test).

**Fig. 8:**
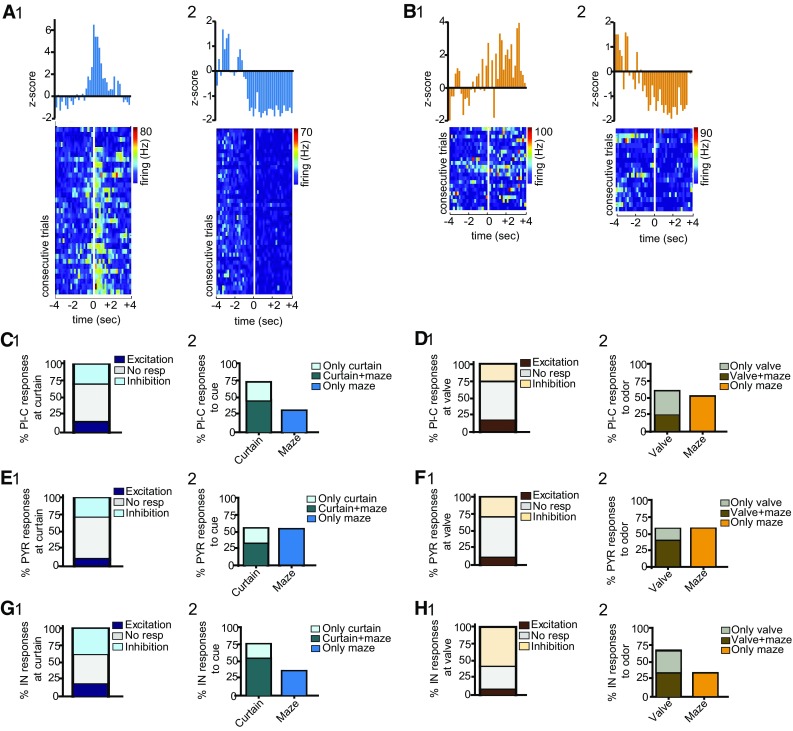
Short-latency responses triggered by crossing of the curtain and activation of the odor valve. **A** 1 Top-event histogram and bottom-normalized firing for one neuron exhibiting excitation at crossing of the curtain (vertical white line); 2 as in 1 but for an inhibitory response; note that, for this cell, changes in firing tended to occur before the triggering stimulus. **B** 1 Example of one excitatory response elicited by valve activation. 2 As in 1 but for one inhibitory response. **C** 1 Typologies of cue-triggered, short-latency responses recorded from place cells. 2 Percent distribution of place cell responses during cue trials; neuronal responses were either recorded as event-triggered (curtain) or measured as rate variations during navigation of the cue corridor (maze), all responses were then combined. **D** 1 As in **C**, but for odor responses; valve = responses triggered by the release of odor through the valve. **E** as in **C**, but for pyramidal cell responses at the curtain. **F** As in **D** but for pyramidal cell responses at the valve. **G** As in C but for interneuron responses at the curtain. **H** as in D but for interneuron responses at the valve. *N* = 8 mice; *n* = 25 PlC; *n* = 90 PYR, *n* = 26 IN

The encounter of odor in a familiar context altered the activity of 50% of group II interneurons, and typically elicited increased rates (Wilcoxon rank sum test; Fig. 7F). Odor also affected the firing of 75% of group III cells. When compared to group II, the distribution of group III responses resulted different, with 25% interneurons exhibiting enhanced firing (Fig. 7F; 7G1, 2), and the remaining cells manifesting decreased activities (Fig. 7F; G3, 4). Nevertheless, the magnitude of the elicited responses resulted similar between the two groups (*p* = 0.56, Mann–Whitney test; 30.0 ± 9.2% increase or 19.4 ± 4.2% decrease). Next, we examined whether the odor-evoked changes in firing might occur in association with a specific place of the corridor. During baseline trials, 56.3% of cells were identified as c1 = c2. Exposure to odor did not affect the firing profile of the recorded interneurons (Fig. 7H). For 70% of cells, variations occurred within the first 10 trials (Fig. 7I1) and the evoked dynamics typically progressed during all odor trials (Fig. 7I2, 3). Noticeably, as observed for place cells, the post-Bs firing of all interneurons reverted to baseline levels (*p* = 0.836, Mann–Whitney test). The experience of an unexpected odor did not affect interneuron firing with respect to theta oscillation (active cycles: *p* = 0.148, Wilcoxon signed rank test; vector angle: *p* = 0.839, permutation test; vector length: *p* = 0.8, Wilcoxon signed rank test).

### Short-latency, novel event-triggered responses in dCA1 during novelty detection

Novelty alters hippocampal activity and initiates a cascade of events which culminates with the activation of downstream structures, including the VTA (Lisman and Grace 2005). Accordingly, if the occurrence of unexpected events is genuinely signaled by dCA1 neurons, our recordings should comprise cells exhibiting short-latency responses. We have demonstrated that the encounters of novel stimuli elicit a reshuffling in network connectivity and pronounced rate variations. However, whether mismatch learning is initiated within dCA1 is currently not known. To address this question, we evaluated the latencies of the event-triggered responses. During cue protocol, approaching of the curtain and crossing into the novel compartment c2 elicited either excitation (Fig. 8A1) or inhibition (Fig. 8A2), and these responses tended to last for a few hundred-milliseconds. For some neurons, the changes in firing preceded the occurrence of the triggering signals, maybe reflecting dCA1 computation of events that, although initially unexpected, became progressive predictable. To assess whether dCA1 neurons might detect the occurrence of odors, we measured the changes in firing during valve activation. Likewise cue, odor evoked both excitatory and inhibitory responses (Fig. 8B1, 2). Next, we examined the activity of the different classes of dCA1 neurons. At crossing of the curtain, 43.6% place cells exhibited short-latency responses, of which 15.6% were excitatory (Fig. 8C1). To provide evidence on a possible dCA1 contribution not only in novelty detection but also encoding, we combined all responses measured during cue trials, i.e. the event-triggered responses and responses measured as changes in firing during navigation of the novel corridors (maze responses). Short-latency responses were assessed in 70% of place cells, of which 45% also exhibited maze responses. The other 30% cells exhibited only changes in maze responses (Fig. 8C2). Similar results were obtained during odor trials. The release of odor triggered short-latency responses in 41.7% of place cells (Fig. 8D1). When all odor responses were combined, 52.6% were classified as event-triggered, and of these, 21% also developed rate variations along the corridor (Fig. 8D2).

The crossing of the curtain evoked short-latency responses in 38.9% pyramidal cells (Fig. 8E1). When combining all cue experiments, we identified 19.4% of ‘pure’ event-triggered responses and 30.1% of mixed responses (Fig. 8E2). Short-latency responses to odor manifested a profile similar to cue, with 40% of significant responses recorded (Fig. 8F1). Moreover, the percent of neurons responding to the release of odor and of neurons exhibiting maze dynamics were comparable (Fig. 8F2), whereas 36.5% of cells manifested mixed responses (Fig. 8F2).

Event-triggered responses were recorded from 58% of interneurons, 38.7% of which were inhibitory (Fig. 8G1). Responses measured at the curtain and during maze navigation were then combined; 67.8% of cells exhibited event-triggered changes, of which 50.0% were identified as mixed responses (Fig. 8G2). Interneurons resulted sensitive also to odor presentation, with 65.4% of inhibitory responses recorded (Fig. 8H1). Although the majority of interneurons expressed short-latency responses, the distribution of responses among all conditions was proportionally similar (Fig. 8H2).

### The encounter of a qualitative different reward evokes rate variations in hippocampus dCA1 neurons

Increasing evidence indicates that hippocampal neurons signal changes in reward location (Dupret et al. 2010). To assess whether dCA1 can detect changes in the valence and in the significance of the rewards, we determined neuronal firing during the experience of rewards with increased incentive salience (see Experimental procedures; Fig. 1C4, C5), which were provided at the same location as the familiar reward. The experience of a different reward did not significantly affect mice behavioral responses. We did not measure variations in the velocity (*p* = 0.272, Two-way Repeated Measure ANOVA), in the traveled space (*p* = 0.344, Mann–Whitney test) or in the time spent to complete each lap (*p* = 0.109, Mann–Whitney test). Analysis of maze occupancy during both pre-stimulus baseline and reward trials yielded to 85.4 ± 1.5 and 82.9 ± 3.9% coverage, respectively.

**Fig. 9:**
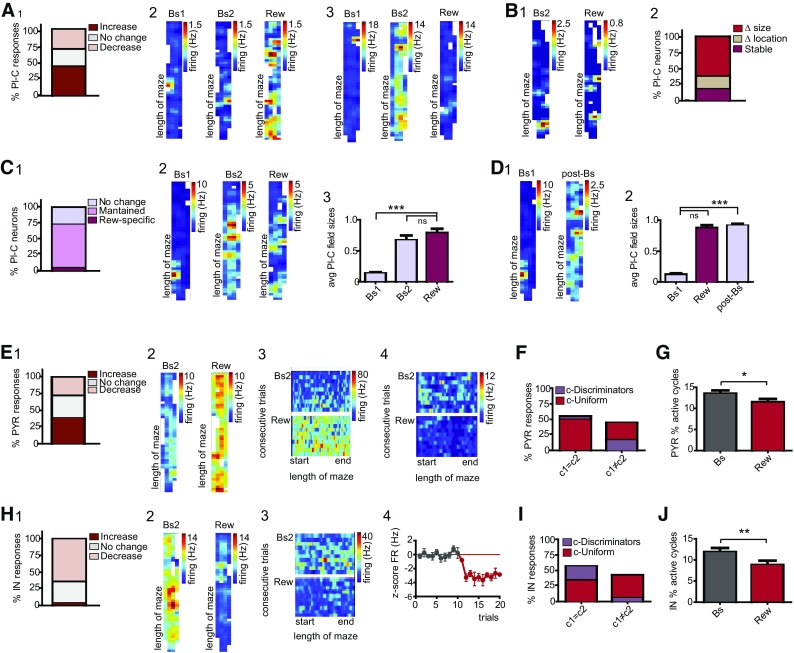
Dorsal CA1 neuronal responses to reward novelty. **A** 1 Distribution of place cell responses during new reward trials. 2 The stimulus-specific increase in firing occurred ‘uniformly’ along the entire corridor. 3 In this cell, the experience of an unexpected reward produced attenuation of firing. **B** 1 Example of the changes in field location elicited by the experience of a novel reward. 2 Different typologies of reward-induced effects on place fields. **C** 1 Reward-elicited effects on group II and III field stability. 2 Exposure to a novel reward did not affect the activity of this place cell. 3 Quantification of the size magnitudes (One-way ANOVA). **D** 1 Firing and field sizes for one place cell during pre- and post-Bs; 2 for all place cells, the magnitude of fields recorded during post-stimulus resulted significantly different from the original fields (One-way ANOVA). **E** 1 Percent of pyramidal responses during navigation in the new reward corridor. 2 Example of reward-evoked increase in firing. 3 Enhanced rate variation for a different neuron, showing rapid onset. 4 Decrease in rate for a different cell. **F** Space-associated dynamics evoked by the encounter of a novel reward. **G** Reward-elicited reduction in active theta cycle (Wilcoxon Rank test). **H** 1 Distribution of interneuron responses to unexpected rewards. 2, 3 Examples of rate decrease for two different neurons. 4 Time-course of the evoked inhibitory responses. **I** Analysis of interneuron space-associated firing during reward trials. **J** Significant decrease in the percent of active theta cycles (Wilcoxon Rank test)

Reward novelty typically occurred following the experience of either odor (group II) or novel cues (group III; Fig. 1C5c). Nevertheless, the experience of an unexpected reward induced additional and stimulus-specific variations (Fig. 9A1–3). Data from the two groups were not different and were combined. We recorded 45% of neurons with enhanced firing (Wilcoxon rank sum test; Fig. 9A1, 2), calculated as 127.5 ± 28.7% change, whereas in other 30% neurons the new reward induced a 47.0 ± 11.5% reduction (Fig. 9B1, 3). To elucidate whether rate variations exhibit space-related proprieties, we measured neuronal firing within the two compartments. During pre-stimulus baseline, 60% of neurons were classified as c1 = c2. The experience of a novel reward induced a significant reorganization in place cell firing. Nevertheless, these dynamics were not associated with any particular compartment or area of the corridor. Analysis of the neuronal activity during post-Bs did not reveal differences in rates between pre- and post-Bs (*p* = 0.346, Mann–Whitney test), suggesting a return toward pre-Bs values at cessation of the novel experience. Likewise, cue and odor, navigation of the reward corridor induced a loss in field stability for 81.0% of place cells (Fig. 9B1). For 19% of neurons, this loss consisted of changes in field locations and, coincidentally, the formation of new fields (Fig. 9B1); remapping was not restricted to the novel compartment or the rewarding area. For the remaining 62% of cells, the loss of field stability correlated to a significant enhancement in sizes (Fig. 9C1–3). Noticeably, subsequent analysis revealed a reward-specific effect only for one neuron. In other words, the observed increases rather occurred during the experience of the other stimuli and were then retained during pre-Bs (Bs1 = 0.1 ± 0.02; Bs2 = 0.7 ± 0.08; NewRew = 0.8 ± 0.07; Bs1 vs. Rew, *F*(2, 36) = 32.38, *p* < 0.0001, One-way ANOVA; Bs2 vs. Rew, *p* > 0.05, One-way ANOVA; Fig. 9C1–3). Field sizes recorded during post-Bs resulted also significantly different with respect to pre-Bs (group II and III combined; Bs1 = 0.1 ± 0.02; post-Bs = 0.9 ± 0.03; *F*(2, 30) = 165.5, *p* < 0.0001, One-way ANOVA; Fig. 9D1, 2). Noticeably, no variations in any of the parameters of theta coupling were measured (vector angle: *p* = 0.554, Permutation test; vector length: *p* = 0.401, Wilcoxon signed rank test; active cycles: *p* = 0.444, Wilcoxon signed rank test). Navigation of the reward corridor altered both group II and group III pyramidal cell activities, leading to similar variations. In 40% of neurons (Fig. 9E1–3), reward elicited a 60.4 ± 9.7% increased in firing. Other 27.1% of cells exhibited a significant attenuation in activities (Fig. 9E1, 4), quantified as 26.8 ± 5.4% change. We then interrogated whether rate variations might occur in association with one specific compartment. Navigation of the reward corridor did not affect the firing profile of the 55.3% of c1 = c2 cells; in contrast, 28% of c1 ≠ c2 cells manifested uniform rate variations along the entire corridor (Fig. 9F). Overall, we measured a reward-elicited increase of c-Uniform neurons (Fig. 9F). At cessation of the stimulus, 47% of cells exhibited different activities with respect to pre-Bs (Bs2 = 5.7 ± 1.2 Hz, Bs3 = 11.3 ± 2.1 Hz; *U* = 142.0, *p* = 0.0195, Mann–Whitney test). Reward novelty significantly reduced the number of active theta cycles (Bs2 = 13.5 ± 0.8, NewRew = 11.5 ± 0.8; *p* = 0.03, Wilcoxon Signed Rank test; Fig. 9G), without affecting either the spike-coupling (vector angle; permutation test, *p* = 0.991) nor the vector strength (*p* = 0.919, Wilcoxon Signed Rank test). The experience of new rewards elicited similar effects in group II and III interneurons (Fig. 9H1). Decreased activity was assessed for 63.6% of cells (Wilcoxon rank sum test; Fig. 9H1–4), and measured as 34.6 ± 4.1% change. Increased rates were hardly observed (Fig. 9H1). New reward elicited a pronounced rearrangement in interneuron firing with respect to space (Fig. 9I), however, variations in neuronal firing were not associated with the novel compartment. At cessation of the reward protocol, the encounter of familiar rewards reverted neuronal firing within pre-Bs levels (pre-Bs vs. post-Bs firing; *p* = 0.15, Mann–Whitney test). Although not affecting interneuron’s spike-coupling to theta oscillation (vector length: *p* = 0.615, Permutation test; vector strength: *p* = 0.871, Wilcoxon Signed Rank test), the delivery of a new reward evoked a significant decrease in the number of active theta cycles (Bs2 = 11.9 ± 0.9, NewRew = 8.8 ± 1.0; *p* = 0.002, Wilcoxon signed rank test; Fig. 9J).

**Fig. 10:**
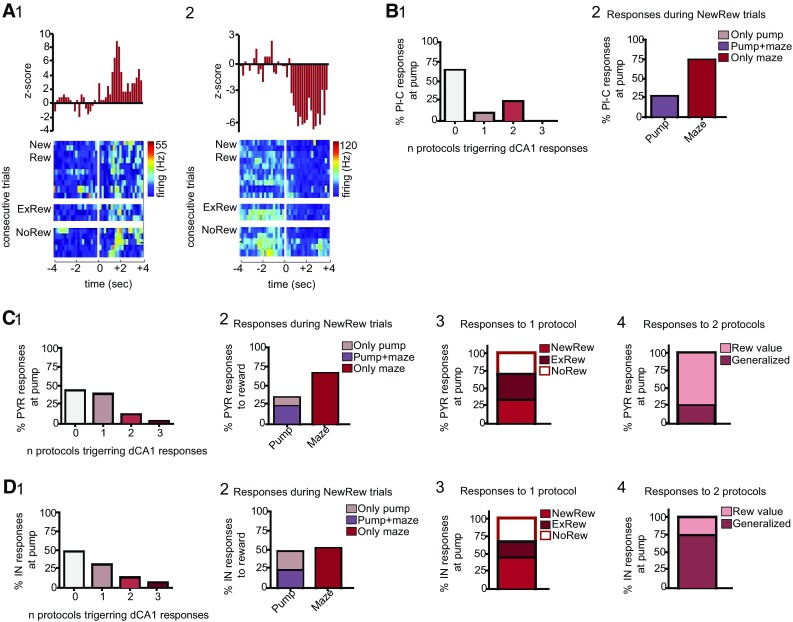
Firing of dCA1 neurons reflects both different reward and the significance of reward experiences. 1 Top- event-histogram for one excitatory response; bottom- event map for the same neuron, note the consistent direction of the evoked-changes during all reward protocols; vertical white line, pump activation. 2 As 1, but for inhibitory responses. **B** 1 Percent distribution of place cell responses during the different reward protocols. 2 Combined responses during new reward protocol, expressed as short-latency responses (pump) and changes in firing associated with the navigation of the reward corridor (maze). **C** 1 As in B1 but for pyramidal cells. 2 As in B2. 3 Response distribution to the three protocols for pyramidal neurons exhibiting only one significant response. 4 As in 3 but for cells responding to two reward protocols. **D** Similar to **C**, but for interneurons

### Dorsal CA1 neurons signal both changes in the valence as well as in the attributed significance of rewards

The role of VTA in reward responses is well-established. A reward, experienced in violation to a subject expectation, with unexpected being either the occurrence or the omission, drive dopamine (DA) neuron into burst firing and evokes DA release in projecting structures (Ljungberg et al. 1992; Schultz and Dickinson 2000). Lisman and Grace (2005) have suggested that, following detection of unexpected events within the dCA1, a signal is conveyed to the VTA through a multi-nuclei path (Floresco et al. 2001, 2003; Goto and Grace 2005). To assess whether dCA1 signal the violation of expected rewards, we examined short-latency responses triggered by the activation of the pump (see Experimental procedures; Fig. 1C5 bottom). First, we assessed event-responses during the experience of qualitative different reward (NewRew). To demonstrate whether dCA1 neurons also detect the significance of the rewarding experiences, we design protocols in which the amount of novel reward was either tripled or omitted (Fig. 1C5, bottom).

Unexpected rewards triggered similar changes in all classes of neurons; these responses displayed short latencies and were either excitatory or inhibitory (Fig. 10A1, 2). For those neurons responding to more than one reward protocol, the direction of the subsequent responses resulted aligned to the first one (Fig. 10A1, 2, bottom). We also observed neurons exhibiting a second response, which typically occurred slightly delayed with respect to pump activation (Fig. 10A1). The experience of unexpected rewards evoked short-latency responses in 35% of place cells, with the majority of neurons responding to two reward protocols (Fig. 10B1). To demonstrate the contribution of these neurons in detection and encoding, we examined the correlation between event triggered- and maze responses recorded during new reward trials (Fig. 1C5b,c). All neurons exhibiting pump-triggered responses also exhibited altered maze dynamics (Fig. 10B2). Place cells resulted similarly susceptible to both the protocols examining the valence of rewards (quality; NewRew) and its significance (quantity; Ex- and NoRew). Reward-triggered responses were assessed in 55.6% of pyramidal cells (Fig. 10C1) and consisted in either excitation or inhibition. All different protocols elicited comparable excitatory responses (*p* = 0.265, One-way ANOVA), estimated as 75.5 ± 22.5% change. In contrast, the magnitudes of inhibitory responses elicited during protocols examining reward values resulted significantly different (NewR: 47.8 ± % 8.9, ExR = 66.6 ± 6.0%, NoR = 37.4 ± 5.8%; *F*(2, 34) = 6.186, *p* = 0.0051, One-Way ANOVA).We identified 38% of neurons manifesting short-latency responses, and the majority of these were mixed responses (short-latency + maze responses). Conversely, most cells exhibited maze-evoked dynamics (Fig. 10C2). For neurons exhibiting only one response, the distribution of the responses to each protocol was comparable; specifically, 36.1% of responses were assessed during extra reward, whereas 30.5% during no-reward trials (Fig. 10C3). For neurons responding to two reward protocols, 72.7% responses were measured during both extra- and no-reward trials, i.e. the protocols examining the significance of the rewards. Conversely, for a smaller population of these cells, one of the two responses was measured during novel reward trials (Fig. 10C4). Thus, we concluded that pyramidal cells manifested the propensity to signal the value of rewarding experiences. Unexpected rewards elicited significant responses in 51.7% of interneurons (Fig. 10D1). All events were inhibitory and their magnitudes were comparable among protocols (*p* = 0.522, One-way ANOVA), with the combined values assessed as 36.0 ± 2.4% change. When all NewRew-responses were combined, the distribution between event-triggered and maze responses resulted similar (Fig. 10D2). Noticeably, we also recorded 23.6% of interneurons manifesting mixed responses (Fig. 10D2). For interneurons susceptible to only one protocol, the distribution of the responses to each protocol resulted similar (Fig. 10D3). On the other hand, 75% of neurons responding to two protocols manifested generalized responses, with at least one significant response recorded during new reward trials (Fig. 10D4).

### Effects of novelty on the dCA1 neuron bursting activity

The firing of cortical neurons can occur in regular- or bursting mode (Ranck 1973). Previous studies have revealed altered dCA1 patterns of activities during space navigation and hippocampal-dependent cognitive tasks (Huxter et al. 2003; McNaughton et al. 2006). To address whether unexpected changes in context might affect the modality of dCA1 neuron firing, we interrogated the effects of novelty on the percent of burst firing (Valenti et al. 2011), on the interspike interval (ISI) and on the number of detected spikes per burst. Despite the pronounced effects on dynamics, the experience of novelty did not affect dCA1 pattern of firing and bursting activity (for all classes of dCA1 neurons and all parameters examined: *p* > 0.05; Mann–Whitney test).

## Discussions

### The reconfiguration of excitation/inhibition balance in dCA1 during the encounter of novel stimuli

Surprising events catch our curiosity and foster the acquisition and storage of information. Within dCA1, the capability to discriminate similarities across different experiences might promote either the excerpt from stored memories or the acquisition of novel information. A hypothesis posits that learning occurs through a computation based on expectation and is initiated when events occur in violation of previous experiences (Vinogradova 2001; Lisman and Grace 2005). Accordingly, we interrogated dCA1 neuronal operation during the experience of unexpected events. We sought to investigate the means by which deputed neurons can discriminate among, and specifically signal, distinct stimuli when these are experienced in sequence and within the same context. Moreover, we aimed at elucidating whether stimuli sharing similar proprieties and possibly activating similar circuits, might induce cross-sensitization and attenuate the subsequent neuronal responses. To this end, we employed acute silicon probe recordings and delivered specific stimuli while mice navigated familiar virtual spaces. During the past years, VR systems have been employed to investigate place cell dynamics (Harvey et al. 2009; Dombeck et al. 2010). A report indicated accurate spatial selectivity and firing rates when place cell activities in VR are compared to recordings in the real world (Ravassard et al. 2013). These data validate the VR approach, as they indicate that distal visual cues and self-motion are sufficient for the establishment of place fields and, therefore, for higher cognitive functions. Noticeably, VR allows an innovative and unique approach to studying novelty, as changes can be promptly delivered within the same context and coincidentally to mice navigation, thus avoiding disruption of behavior. For the investigation of novelty in the real world, baseline activities recorded in one context are typically compared to data collected following the physical relocation of subjects into fairly different contexts. Thus, subjects do not have an online parameter of comparison. Noticeably, in some cases, the calculation was carried by comparing data collected at different times or even different days. This experimental design might raise questions on whether the outcome of these experiments is actually reflecting a computation in which the incoming information is matched to stored memories. If the encoding of experiences within hippocampus is indeed dependent upon match/mismatch computation, this issue became of critical relevance. In our design, novel stimuli were embedded in otherwise familiar contexts, and delivered in one specific compartment, whereas the second compartment retained features of the familiar corridor. Due to a visual impediment positioned inside the corridor, a portion of the environment resulted hidden to the subjects’ view, and this design possibly helped in promoting a dCA1-dependant comparison between the two compartments. Noticeably, this configuration recapitulates features of the tasks employed in human experiments (Duncan et al. 2012) and should allow studying match/mismatch computation on mice. Indeed, human studies have shown a pronounced and subfield-specific activation of dCA1 when portions of the environment are familiar, i.e. likely to elicit expectation, and contingent with unexpected stimuli (Köhler et al. 2005; Kumaran and Maguire 2006a, 2007b). Therefore, elucidating dCA1 dynamics would ultimately facilitate the correlation between electrophysiological measurements in rodents and the increase in hippocampal signal observed in humans. The encounter of different unexpected stimuli was signaled by dCA1 neurons through variations in firing rates. Novelty-evoked responses were not generalized; rather, most neurons appeared to discriminate among events of different nature. Previous experiences of stimuli sharing similar properties, or possibly activating convergent inputs, orchestrated the subsequent response of principal cells and induced cross-sensitization.

Hippocampal place cells signal the passages of a subject through one specific place of the environment (O’Keefe and Dostrovsky 1971; O’Keefe and Nadel 1978; Maren and Holt 2000). This pattern is not immutable but shaped by circumstances and behavior. In pronouncedly different environments, new fields of the place cell can emerge, and established ones disappear or relocate, in accordance with a process termed global remapping (Muller and Kubie 1987; Colgin et al. 2008). Conversely, minor or progressive variations in context elicit changes in firing, or ‘rate remapping’ (Leutgeb et al. 2005). Thus, place cells are not simple space-detectors, rather they support several hippocampal-dependent higher cognitive functions (Eichenbaum 2004; Kentros et al. 2004; Colgin et al. 2008; Eichenbaum and Cohen 2014). In our study, navigation of novel corridors promoted reorganization in place cell firing, which unfolded into unspecific activities along the entire maze. The experience of a different reward also elicited rate variations that resulted not associated with a specific place of the corridor. On the other hand, odor-evoked dynamics occurred within definite sectors, including the sector where the odor was experienced. We postulate that these changes might reflect a loss of structured firing implemented to facilitate the acquisition of novel information. In this respect, dCA1 dynamics, elicited by the encounters of unexpected stimuli, might promote both the comparison and the integration of information carried by dCA3 and EC. Firing dynamics also promote the discriminative encoding of different stimuli, providing a mean to avoid interference across similar memories (Quirk et al. 1990; Anderson and Jeffery 2003; Wills 2005; Colgin et al. 2008). Noticeably, dCA1 neurons receive inputs from both these regions. Variations of dCA1 firing favor the integration of sensory information conveyed by the EC (Hafting et al. 2005; Rennó-Costa et al. 2010). The extensive recurrent collaterals of CA3 permit the retrieval of stored information (McNaughton and Morris 1987a; Lisman 1999). Within the dCA1, EC and CA3 inputs are then integrated and the information might be compared in accordance with a match/mismatch computation (Vinogradova 2001). As a matter of fact, a recent investigation demonstrated that dCA1 neuron spike-timing is governed by a CA3-EC dual input (Fernández-Ruiz et al. 2017).

Global and rate remapping are typically acknowledged as different facets of the same language employed by the hippocampus to warn against changes that require behavioral adaptation (Colgin et al. 2008). The propensity to express one code or the other might reside on the differences between two contexts, in other words, how novel the stimuli are upon subjective computation (Muller 1996). During odor or reward trials, we observed that the original fields of ~ 20% of place cells disappeared in coincidence to the actualization of novel fields. Odor-evoked remapping typically occurred within the novel compartment. Conversely, the establishment of new fields during reward trials resulted not associated with any specific compartment or with the rewarding area. Thus, the observed remapping might account for a subjective response to an environment recognized as different. Alternatively, changes in field location might reflect a parallel computation, and guide associative learning.

Novel stimuli experienced in familiar contexts also altered pyramidal cell and interneuron’s firing. When encountered at first, unexpected events mostly increased the rates, although a proportion of neurons with decreased activity was also detected. The co-occurrence of inhibition might emphasize the disinhibition of neighboring cells, and enhance the saliency of the detected signals. On the other hand, given that mostly occurred during the last stimulus, the inhibitory responses might be of critical importance to avoid interference across events or to dampen the established networks in order to facilitate the reconfiguration of assemblies and re-learning. Together with place cells, variations in other classes of dCA1 neurons might favor the continuous comparison between online stimuli and stored events. Noticeably, increased activities in dCA1 are consistent with the overall activation reported in both human and animal studies during hippocampal-dependent learning tasks (Köhler et al. 2005; Kumaran and Maguire 2006a). In this respect, Pascual-Leone and colleagues employed transcranial magnetic stimulation to interrogate the human cortical motor maps during implicit and explicit learning. They observed a progressive enhancement of the area corresponding to the muscles in use, which reverts to baseline when learning was established (Pascual-Leone et al. 1994). The VTA operates through changes in DA neuron activity during prediction error computation (Ljungberg et al. 1992). Other reports indicate that, in cortical and limbic structures, disinhibition typically occurs in face of events that require attention and culminate in the enhanced activity of projecting neurons (McNaughton and Morris 1987b; Brioni et al. 1989; Arolfo and Brioni 1991; Harris and Westbrook 1995; Knierim et al. 1998; Letzkus et al. 2011, 2015; Caroni 2015). Firing variations appears, thus, as a general modus operandi of different brain structures. We propose that the reconfiguration of dCA1 dynamics, across different networks of neurons, constitutes a biological correlate of learning, and supports cognitive processing.

### Detection of novelty occurs through the recruitment of neighboring networks, and a computation based on expectation

Together with changes in firing, the transition among different cell ensembles also advances cognitive processing (Martin and Morris 2002; Buzsáki 2010). Dupret and colleagues (2010) showed that a reorganization of hippocampal neuronal connections occurs during goal-directed spatial learning. Accordingly, we observed that both the number and the arrangement of dCA1 interactions changed during navigation in unexpected contexts. Neurons appeared to engage in an interdependent computation where the loss of previous interactions ‘makes space’ to the establishment of novel ones. The flickering in connectivity, together with rate variations, would then foster the internal representation of plastic contexts. Noticeably, unexpected events induced changes in neuron interactions that manifested temporal properties similar to the ones assessed for dCA1 dynamics. Consistent with the hypothesis of dCA1 as a detector of novelty, we would expect that the effects on dynamics/connectivity arise within a similar time window and during the first encounters of novel stimuli. In other words, rate variations occurring within the first 10 trials might reflect ‘encoding’. On the other hand, variations arising during the following trials might be associated with ‘habituation’ to a stimulus perceived as no longer unexpected. For the majority of recorded neurons, changes in rates and network connectivity occurred within the first trials and progressed with specific patterns. The rapid onset observed in some responses probably reflects variations being initiated at the recognition of the novel stimuli. The majority of cells manifested a pronounced rearrangement in interactions during the following trials. The different profiles of the time-courses might reflect distinct computation during the experience of novelty. Rate variations that progressed with the task might express a continuous computation between incoming information and extracted memories. Responses that reach plateau could indicate accommodation to a stimulus that might be perceived as no longer unexpected. Peak responses might alert to any sudden change in the environment. Conversely, the measured differences in rates between pre- and post-stimulus baselines might also be regarded as failures in expectation, with unexpected being, in this case, the re-encounter of familiar context after the experience of novelty.

### Event-triggered responses appoint the hippocampal dCA1 as an important structure in novelty computation

Novelty-elicited dynamics might either reflect the computation of deputed neuron/network within the dCA1, be induced by changes occurring in efferent structures, or a combination of the above. In this respect, recording of short-latency responses would be in line with the role of dCA1 neurons in novelty detection as well as with the initial computation of novelty. Conversely, changes in rates occurring during a longer time scale might represent a neuronal adaptation and foster the integration of inputs from different structures. Although most studies agree that novelty is detected within the hippocampus, the actual locus (i.e. the exact subfield) of novelty detection is a matter of debate. By examining event-triggered responses, we sought to shed light on the temporal activation of dCA1 neurons in respect to the occurrence of novel stimuli. The short-latencies of the response to either unexpected cues or odor are consistent with the detection of novelty being computed by dCA1 neurons. For other cells, firing variations took place prior to the occurrence of novel stimuli, in agreement with a reported engagement of dCA1 in the anticipation of novelty (Wittmann et al. 2007). These findings are consistent with previous investigations in rats, reporting event-triggered responses in dCA1 during the processing of novel and unexpected events (Ruusuvirta et al. 1995; Brankačk et al. 1996; Grunwald et al. 1998; Fyhn et al. 2002). Noticeably, a high-resolution fMRI study on human indicates the dCA1 as the only locus of novelty detection among all hippocampus subfields (Duncan et al. 2012), and other evidence builds in supporting the role of dCA1 in match/mismatch learning (Köhler et al. 2005a; Kumaran and Maguire 2006b, 2009; Duncan et al. 2012a). The double activation measured as event-triggered responses and variations in maze dynamics is intriguing and might reflect the engagement of dCA1 neurons in both detection and encoding. Thus, following detection, the processing of the unexpected events might be carried by the altered firing along the maze. In other words, deputed dCA1 neurons might initiate a cascade of alterations that develops through pronounced rate variations. Subsequently, these changes might be transferred to neighboring neurons and promote the progressive recruitment of different dCA1 networks in order to facilitate input integration and learning.

Evidence suggests that dCA1 neurons are involved in processing reward experience and signal the changes in reward location. However, these responses might be secondary to VTA activation. Thus, it is not known whether dCA1 neurons can detect the occurrence of rewards with enhanced valence nor if they can signal rewarding events holding different significance. In view of the hypothesis that hippocampus and VTA constitute a functional loop, this question is fundamental. Indeed, the ability to perceive rewarding events would place the hippocampus as a detector of stimuli carrying incentive motivational properties and reveal a novel role in signaling the violation of reward expectation. To test this hypothesis, we design a set of experiments that recapitulated rewarding processes previously reported to activate VTA DA neurons and to promote prediction-error computation (Schultz and Dickinson 2000). Rapid changes in rates were assessed at the delivery of both unexpected qualities and quantities of rewards, indicating a compelling role to the dCA1 in detecting both the valence and significance of rewarding experiences. The majority of cells exhibiting short-latency responses during new reward trials also manifested firing dynamics associated to the navigation of the maze, suggesting the engagement of dCA1 neurons in both detection and encoding of novel rewarding experiences. Taken together, these data are also in accordance with studies indicating that the target areas of dopaminergic projection express value-related signals. The experience of unexpected rewards enhances the coordinated reactivation of dCA1 place cells during SWR (Singer and Frank 2009; Ambrose et al. 2016). A failure in the quantitative estimation of expected outcomes also elicits pronounced activation in the dCA1 (Lee et al. 2012). Human studies revealed a coincident activation of VTA and hippocampus during motivated learning (Adcock et al. 2006). The capacity of dCA1 neurons to signal a violation in expected reward might, therefore, parallels - or maybe even drives - DA neuron responses during prediction error coding, and would be consistent with the assessed functional alliance between the hippocampus and VTA.

### The cognitive implications of surprising events

A framework for the encoding of novelty seems to be emerging. In dCA1, sensory stimuli are matched to previous knowledge. If events occur in violation of subject expectations, hippocampus activity is enhanced and an alerting message is triggered to the VTA, which in turns elicits arousal of the DA system. Our data provide further support to the role of dCA1 in match/mismatch learning and might contribute to shed light on the functional alliance between hippocampus and VTA during higher cognitive functions.

In this respect, by employing animal models, a recent report has investigated the implication of DA in Alzheimer disease and revealed an age-dependent loss of DA neurons in the VTA, but not substantia nigra compacta, which preceded the advancement of the characteristic plaques in hippocampus (Nobili et al. 2017). Hyperactivity within CA1 hippocampus, and associated disruption of VTA DA neuron function has also been demonstrated in animal model of schizophrenia and stress-associated disorders (Lodge and Grace 2011; Valenti et al. 2011). Accordingly, energy deficit in parvalbumin neurons, due to mitochondrial dysfunction, affects both network physiology and connectivity, resulting in impaired sensory gating, motor and social disabilities (Inan et al. 2016; Kann 2016). Energy failures elicit also waives of mass depolarization, which can lead to stroke and migraine (Dreier and Reiffurth 2015). Last, but not least, a recent investigation has revealed a loss of coordination in dCA1 assemblies’ discharge in a mouse model of Fragile X, which the authors suggest might correlate to the intellectual disabilities observed in humans (Talbot et al. 2018). In summary, we envision that a deeper understanding of the hippocampus-VTA loop and annexed circuitry will shed light on cognitive functions as well as on the mechanism underlying several neurological and psychiatry disorders.

## Electronic supplementary material

Below is the link to the electronic supplementary material.

Supplementary material 1 (EPS 7477 KB)

Supplementary material 2 (DOCX 10 KB)
