# Supplementary material for: The cognitive nuances of surprising events: exposure to unexpected stimuli elicits firing variations in neurons of the dorsal CA1 hippocampus
Source: Brain Struct Funct. 2018 May 22;223(7):3183–211. doi: 10.1007/s00429-018-1681-6 (PMC6132666; doi:10.1007/s00429-018-1681-6)
Supplement: Supplementary file 2 — Supplementary material 2 (DOCX 10 KB) [file 429_2018_1681_MOESM2_ESM.docx]

**Supplementary figure 1**

**A**. 1- Distribution of pyramidal and place cell responses elicited by the exposure to novel cues. Each bar summarizes the percent of responses recorded from different mice. 2,3- As in 1 but for responses to odor and reward, respectively.

**B**. Normalized maps of the novelty-elicited increases in firing. The examples reported show similar profiles in responses across different novel stimuli (stimuli are indicated on the side) and different experiments. Numbers (1-8) indicate to the mouse from which each neuron was recorded. Only pyramidal and place cell responses are reported. Specific neurons are indicated on top of the maps by the number of the tetrode-contact (t) and the number of unit (u) assigned to the neuron during clustering.

**C**. As in B, but for decreased responses.
